# Nitrogen cycling and microbial cooperation in the terrestrial subsurface

**DOI:** 10.1038/s41396-022-01300-0

**Published:** 2022-08-08

**Authors:** Olivia E. Mosley, Emilie Gios, Murray Close, Louise Weaver, Chris Daughney, Kim M. Handley

**Affiliations:** 1 School of Biological Sciences, The University of Auckland, Auckland, New Zealand; 2 Institute of Environmental Science and Research, Christchurch, New Zealand; 3 National Institute of Water and Atmospheric Research, Wellington, New Zealand

**Keywords:** Freshwater ecology, Environmental chemistry, Microbial communities, Water microbiology

## Abstract

The nitrogen cycle plays a major role in aquatic nitrogen transformations, including in the terrestrial subsurface. However, the variety of transformations remains understudied. To determine how nitrogen cycling microorganisms respond to different aquifer chemistries, we sampled groundwater with varying nutrient and oxygen contents. Genes and transcripts involved in major nitrogen-cycling pathways were quantified from 55 and 26 sites, respectively, and metagenomes and metatranscriptomes were analyzed from a subset of oxic and dysoxic sites (0.3-1.1 mg/L bulk dissolved oxygen). Nitrogen-cycling mechanisms (e.g. ammonia oxidation, denitrification, dissimilatory nitrate reduction to ammonium) were prevalent and highly redundant, regardless of site-specific physicochemistry or nitrate availability, and present in 40% of reconstructed genomes, suggesting that nitrogen cycling is a core function of aquifer communities. Transcriptional activity for nitrification, denitrification, nitrite-dependent anaerobic methane oxidation and anaerobic ammonia oxidation (anammox) occurred simultaneously in oxic and dysoxic groundwater, indicating the availability of oxic-anoxic interfaces. Concurrent activity by these microorganisms indicates potential synergisms through metabolite exchange across these interfaces (e.g. nitrite and oxygen). Fragmented denitrification pathway encoding and transcription was widespread among groundwater bacteria, although a considerable proportion of associated transcriptional activity was driven by complete denitrifiers, especially under dysoxic conditions. Despite large differences in transcription, the capacity for the final steps of denitrification was largely invariant to aquifer conditions, and most genes and transcripts encoding N_2_O reductases were the atypical Sec-dependant type, suggesting energy-efficiency prioritization. Results provide insights into the capacity for cooperative relationships in groundwater communities, and the richness and complexity of metabolic mechanisms leading to the loss of fixed nitrogen.

## Introduction

Groundwater represents the largest accessible freshwater source on Earth and is stored in permeable geological units known as aquifers that are generally characterized by long water residence times, low organic matter, and slow water exchange rates [1–3]. Natural stores of inorganic nitrogen (nitrate, nitrite, ammonium) are typically present in low concentrations [4]. However, long residence times and close links to surface water (e.g., lakes, rivers, and wetlands) make groundwater susceptible to pollution from nitrogen-based fertilisers [5]. Nitrogen (N) contamination in the terrestrial subsurface has become a global problem [6–8], presenting health risks associated with nitrate in drinking water, such as methaemoglobinaemia and cancer [9], along with eutrophication of surface waters [10].

Groundwater microbial communities contain extensive phylogenetic novelty [11]. While the metabolic capabilities of many groundwater microorganisms remain to be tested in laboratory conditions, genetic evidence suggests that a diverse collection of bacteria and archaea transform nitrogen in groundwater, including novel candidate phyla [12], diverse bacterial taxa [11, 13], archaeal ammonium oxidizers [13, 14], and novel aquifer-adapted clades of anammox bacteria [15]. Research also suggests that numerous aquifer organisms are equipped with genes encoding partial nitrogen cycle pathways, such as nitrite reduction [11, 16]. This has been shown to be a common feature of bacteria and archaea from other habitats [17], and suggests that cooperative interactions are commonly employed to complete individual nitrogen-cycling pathways.

The microbial nitrogen cycle comprises six distinct N-transformation processes, including ammonification, nitrogen fixation, nitrification, denitrification, anaerobic ammonium oxidation (anammox), and assimilation [18, 19]. Microorganisms that perform these processes can be sources and sinks of nitrate. Despite distinct requirements (e.g. for oxygen), many reactions in the nitrogen cycle tend to co-occur in the environment, leading to efficient nitrogen recycling [19], competition for the same resource (e.g. by respiratory ammonifiers and denitrifiers [20]), cooperative completion of the modular denitrification pathway [19], and coupled processes, such as nitrification—denitrification [21] or nitrification—anammox [22]. Accordingly, biological processes derived from networks of microorganisms in the terrestrial subsurface play a dominant role in N-transformations [23].

Denitrification is the most studied nitrogen cycling process in groundwater to-date, due to its importance for nitrogen pollution removal [5], although operation of the truncated pathway produces less desirable forms of inorganic nitrogen - NO_2_^-^ due to its toxicity [24], and greenhouse gases NO and N_2_O [5]. Microbial denitrification is typically linked to dissolved organic carbon concentrations in aquifers, but is also fuelled by inorganic electron donors, such as reduced forms of iron or sulfur [5, 25]. Inorganic donors may be the primary source of electrons for nitrogen-cycling taxa given widespread organic carbon-limitation in aquifers [26, 27]. Accordingly, nitrification and anammox appear to be typical features of shallow oxic or partially oxic aquifers [15, 26], and carbon-limitation can create an opportunity for anammox to outcompete denitrification [28]. However, the occurrence of, or capacity for, these processes may not to be ubiquitous. A scarcity or lack of organisms capable of some processes, including nitrogen fixation, ammonia oxidation, and nitrous oxide reduction, has been reported from one low-oxygen aquifer [11]. Further work is needed to determine the distribution of nitrogen-cycling processes across different aquifers, including aquifers defined based on redox conditions and nutrient characteristics, such as pristine or N-contaminated.

This study investigates the microbial nitrogen cycle in aquifers traversing a wide range of nitrogen, organic carbon, and oxygen concentrations. We determined the metabolic capacity for each pathway in oxic and dysoxic groundwaters, and the transcriptional activity associated with these pathways (understudied in aquifers due to low cell densities) [29]. As aquifers comprise both suspended and attached communities, with distinct compositions and capacities for biogeochemical cycling [30], analyses included both groundwater (planktonic fraction) and groundwater enriched with the sediment-attached fraction. To further characterize reactions leading to nitrogen loss, we quantified ammonia monooxygenase (archaeal and bacterial ammonia oxidation), nitrous oxide reductase (final step in denitrification), and hydrazine synthase (anammox) genes in 64 groundwater samples (from 59 wells) and transcripts in 26, collected up to 860 km apart. Results give insights into environmental factors influencing the presence, co-occurrence, and transcriptional activity of nitrogen-cycling mechanisms, which determine the fate of nitrogen in aquifers.

## Materials and methods

### Study sites and sample collection

Eighty samples were collected from 59 wells, spanning 10 aquifers (mostly sandy-gravel) in the Auckland, Waikato, Wellington, and Canterbury regions, New Zealand (Fig. S1; Table S1). Wells were 4.5–114.6 m deep (18.9 m on average, Table S1). Wells were purged (~3–5 borehole volumes). Then, 3–90 L of groundwater (67 samples) or 0.5–15 L of attached-fraction enriched groundwater (13 samples, Canterbury sites A–D) were collected and immediately filtered on-site. The biofilm or “attached” fraction enriched groundwater (i.e combining planktonic and biofilm aquifer fractions) was collected directly following standard groundwater collection. Prior to collection of these samples, a low-frequency custom sonicator, as described by Close et al. [31] (2.43 kW) was applied for 2 min to detach biofilms and particles from the surrounding aquifer. Biomass was captured onto 142 mm diameter mixed cellulose ester membrane filters (1.2 µm pore size pre-filter over a 0.22 µm filter) using a 142 mm stainless steel filter holder (Merck Millipore Ltd, Cork, Ireland). Both filters were immediately submerged in RNAlater (ThermoFisher Scientific, Waltham, MA, USA), transported on dry ice, and stored at −80 °C. All samples were used to generate amplicon data (×80). A subset was used to quantify functional genes (×64) and transcripts (×26). A subset from Canterbury was used for metagenomics (×16) and metatranscriptomics (×6).

Dissolved oxygen (DO), water temperature, pH, oxidation-reduction potential (ORP), and specific conductance were collected on site using flow-through cell and field probes (YSI EXO sonde 2, YSI PRO+ and YSI ProDSS, Yellow Springs, OH, USA). Samples were grouped into categories based on DO concentrations: anoxic (0 mg/L), suboxic (<0.3 mg/L), dysoxic (0.3–3 mg/L or 9.4–93.8 μM), and oxic (>3 mg/L) as previously proposed for groundwater [32]. Unfiltered groundwater samples were analyzed for P, N, C, S, Fe, Cu, and alkalinity at Hill Laboratories (Hamilton, New Zealand; Supplementary Information).

### Nucleic acid extraction, sequencing and genome assembly

Nucleic acid extraction for droplet digital PCR (ddPCR), 16S rRNA gene amplicons, metagenomes, metatranscriptomes, sequencing, metagenome-assembled genome generation, and transcript mapping, are as described previously [15], and detailed in the Supplementary Information. Only RNA samples with RIN ≥ 6 or DV200 > 30% (i.e. fragments >200 nucleotides) were used for downstream analyses. Metagenome-assembled genome (MAG) completeness and contamination were estimated using CheckM v1.0.12 [33], and MAGs were classified using the Genome Taxonomy Database taxonomic classification tool, GTDB-Tk v0.2.1 [34]. Metatranscriptomic reads were mapped to MAGs using Bowtie2 [35] (v2.3.5, --end-to-end --very_sensitive). Read counts were determined using featureCounts [36] (v1.6.3, -F SAF). Singleton mapped reads per gene were removed. Read counts were normalized to a modified version of transcripts per kilobase per million reads mapped (modified-TPM) [37] via (number of reads mapped to gene)*(1000/gene length)*(1000000/library size).

### Quantitative PCR

Droplet digital PCR (ddPCR) of hydrazine synthase (*hzsB*), ammonia monoxygenase (*amoA*), *nosZ* clade I genes, and transcripts used the QX200 platform with 20 µl reactions, 10 µl 2× EvaGreen Supermix (Biorad, Hercules, CA, US), and 1 µl DNA or cDNA. RNA (24 pg–1.1 µg) was converted to cDNA using Superscript III Supermix (Invitrogen). DPEC-treated water was used for negative controls and gBlock dsDNA fragments were used as positive controls (Table S2; Integrated DNA Technologies, Coralville, IA, USA). Primers and PCR conditions are described in Table S2. Data were analyzed using the QuantaSoft software package v1.0 (Bio-Rad). Positive droplet thresholds were set based on negative and positive droplet fluorescence amplitudes using the positive control as reference.

### Metabolic predictions

Protein-coding gene sequences from MAGs and metagenomic reads were predicted, and annotated (Supplementary Information Table S3). Phylogenetic trees for HzsABC (see figure 8 in Mosley et al. [15]), AmoA, PmoA, NosZ, and NxrA were generated by aligning predicted protein (amino acid) sequences using MUSCLE with default parameters [38], and constructing trees using FastTree (for AmoA) or IQ-TREE (for others) with 1000 bootstraps [39, 40]. IslandViewer4 [41] was used to identify genomic islands in MAG nzgw5.

### Statistical analyses

Significant environmental factors were determined using R package vegan v2.5.6 functions (metaMDS, adonis, vegdist, and envfit) [42]. Bray-Curtis dissimilarities were constructed using vegan with relative abundances of MAGs or protein-coding sequences in metagenomic reads [42]. Heatmap Z-scores were calculated using Heatmap.2 from the gplots package [43]. Linear discriminant analysis and Kruskal–Wallis tests were determined using the Galaxy computational tool (http://huttenhower.sph.harvard.edu/galaxy/). Adonis permutation tests were undertaken using Bray-Curtis dissimilarities. Spearman’s rank correlations (*r*) were calculated using ‘rcorr’ from the Hmisc package, and *p* values were adjusted using the Benjamini-Hochberg method. Statistical analyses were considered significant with *p* < 0.05.

## Results and discussion

### Distribution of nitrogen-cycling pathways in groundwater

#### Differences in nitrogen-cycling processes based on oxygen and nitrate concentrations

**Fig. 1:**
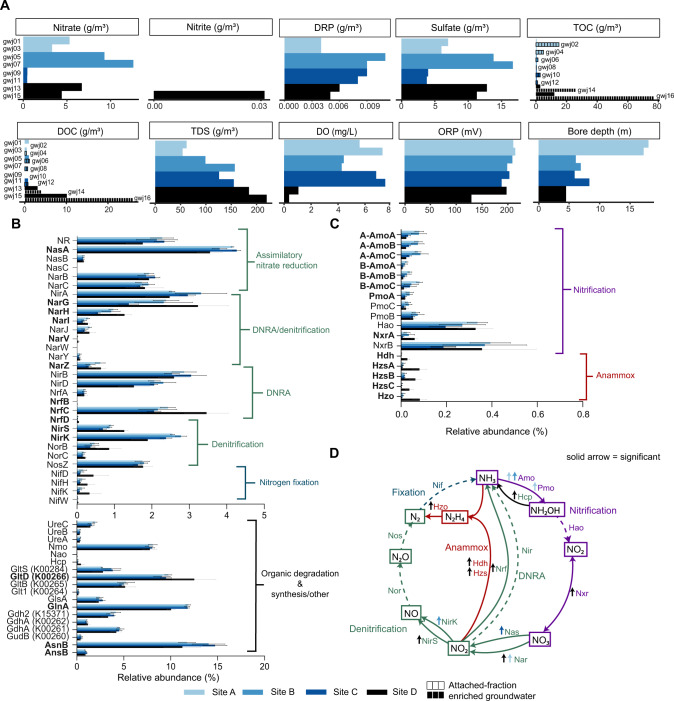
Geochemistry and protein-coding sequences (based on reads) involved in nitrogen cycling that are significantly different among sites used for metagenomics. **A** Bar plots showing geochemical data from groundwater samples, coloured according to site. Solid bar colour = groundwater samples. Grid lines = attached-fraction enriched groundwater. All samples from site D were characterized as dysoxic, although gwj15-16 contained 0.37 mg/L DO, which are near suboxic levels (i.e. <0.3 mg/L). For all samples shown, ammoniacal-N values were below detection. **B**, **C** Bar plots showing the abundance (average of four wells per site and standard deviation) of sequence reads encoding dissimilatory and assimilatory nitrogen-cycling proteins relative to all nitrogen-cycling processes. Predicted proteins that were statistically different between sites are bolded (*y*-axis). **D** Schematic of the nitrogen cycle displaying statistically significant differences between sites (LEfSe, Kruskal–Wallis test, *p* < 0.05). Solid lines depict pathways that were significantly more abundant across the sites, whereas dashed lines indicate no significant difference. Arrows indicate the site with significantly more genes. Abbreviations: A-Amo Archaeal Amo, B-Amo Bacterial Amo, Amo ammonia monooxygenase, Pmo Particulate methane monooxygenase, Hao hydroxylamine oxidoreductase, Nxr nitrite oxidoreductase, Nar nitrate reductase (dissimilatory), Nas nitrate reductase (assimilatory), NirK copper-containing nitrite reductase, NirS cytochrome *cd*_1_-containing nitrite reductase, Nor nitric oxide reductase, Nos nitrous oxide reductase, Nif nitrogenase (various), Hcp hydroxylamine reductase, Nir NADPH-nitrite reductase, Nrf nitrate reductase (associated with Nap), Hdh hydrazine hydrogenase, Hzs hydrazine synthase, Hzo hydrazine oxidoreductase.

Sixteen metagenomes (Table S4) were obtained from duplicate wells at four sites (A–D) from two unconfined alluvial aquifers (Canterbury, Fig. S1). These sites encompassed varied nitrate (0.45–12.6 g/m^3^), DO (0.37–7.5 mg/L), and dissolved organic carbon (DOC) (0–26 g/m^3^) concentrations (Fig. 1A; Table S1). Nitrate concentrations were pristine (site C) to N-contaminated (sites A, B, D) [4]. Sites A–C were oxic and had low DOC (typical of groundwaters), whereas site D was dysoxic with relatively high DOC. Metagenomes from groundwater wells comprised pairs, representing the planktonic and sediment-attached fractions. Over 70 Gbp of raw sequence was generated per site (390 Gbp overall, 322 Gbp trimmed). However, <10% of trimmed reads per sample assembled into contigs >2Kb long and only 0.64–8.14% of reads (3.8% on average) mapped to MAGs (Table S4), reflecting the complexity of microbial communities in the terrestrial subsurface [11]. To capture this diversity, metagenomic reads are first used here to determine the distribution of N metabolisms.

A total of 4,462,950 sequences encoding nitrogen-cycling proteins were identified from metagenomic reads (1,013,871–1,211,591/site, Table S5). The relative abundance of 29/68 nitrogen-cycling gene (sub)families in NCycDB [44] differed significantly among sites (Fig. 1B, C; LEfSe, Table S6). Most of these encoded nitrification and anammox pathways (*p* < 0.05). Sequences encoding other pathways, such as nitrogen fixation and the final steps of denitrification remained consistent across sites (*p* > 0.05).

**Fig. 2:**
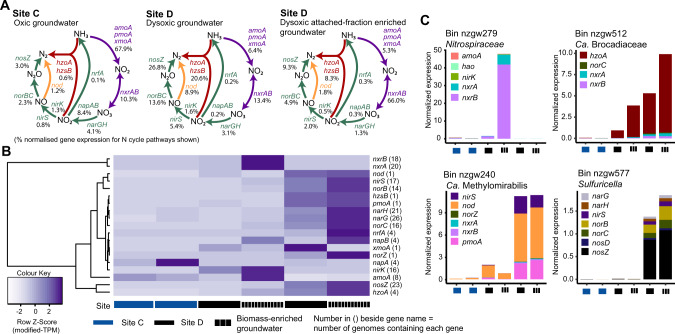
Nitrogen-cycling gene transcription at site C groundwater and site D groundwater and attached-fraction enriched groundwater. **A** Nitrogen cycle schematics display the average abundance of nitrogen-cycling transcripts (based on modified-TPM values) per site and sample type (relative to nitrogen-cycling pathways overall (as shown). The percentage of gene transcripts associated with each pathway component is shown in black font. Coloured arrows represent pathways (purple = nitrification, green = denitrification and red = anammox). Only NrfA and not NirBD are shown for the DNRA pathway. **B** Heatmap shows nitrogen-cycling modified transcripts per million (modified-TPM) at each site (ordered gwj9, gwj11, gwj13-gwj16), scaled by row (Z-Score). Solid coloured blocks represent groundwater, black grid blocks represent the attached-fraction (or biomass) enriched groundwater. **C** Stacked bar plots display four active nitrogen-cycling genomes and the relative abundance (modified-TPM normalized to genome coverage) of their nitrogen-cycling gene transcripts across each site. Abbreviations: *amo* ammonia monooxygenase, *pmo* particulate methane monooxygenase, *xmo* copper-containing membrane monooxygenase, *nod* nitric oxide dismutase, *nxr* nitrite oxidoreductase, *nar* nitrate reductase (dissimilatory), *nap* periplasmic nitrate reductase, *nirK* copper-containing nitrite reductase, *nirS* cytochrome *cd*_1_-containing nitrite reductase, *nor* nitric oxide reductase, *nos* nitrous oxide reductase, *nrf* nitrate reductase, *hzo* hydrazine oxidoreductase, *hao* hydroxylamine oxidoreductase.

Results indicate the presence of ecologically robust mechanisms for nitrogen removal under the reduced DO conditions of dysoxic site D. In addition to the presence of metagenomic sequences encoding complete nitrification-denitrification, the relative abundances of sequences encoding anammox genes were all significantly higher at this site (LEfSe, Kruskal–Wallis test, *p* < 0.05). Sequences encoding genes involved in nitrate reduction to nitrite (*narGHI*), and those encoding mechanisms for nitrite reduction in the dissimilatory nitrate reduction to ammonia (DNRA) (*nrfBCD*), and denitrification (*nirS*) pathways were also higher. Anammox converts NH_4_^+^ and NO_2_^-^ into N_2_ [45] and can be fuelled by ammonium replenished by DNRA, while anammox and denitrification (coupled to nitrification) represent alternative N_2_-generating pathways. Transcriptomic data showed a relatively high proportion of N-cycling gene expression derived from both anammox (hydrazine synthesis and oxidation) and denitrification (nitrous oxide reduction) in dysoxic groundwater (higher than that indicated by gene relative abundances), and showed that nitrifiers were transcriptionally active (Fig. 2A, B).

Oxygen availability (oxic vs suboxic/anoxic) is linked to archaeal and bacterial ammonia-oxidizer abundance or absence in aquifers [11, 26]. As may be expected for an aerobic process, there were significantly more metagenomic sequences encoding the first step of nitrification (bacterial and archaeal *amoABC*) at oxic sites A and B (LEfSe, Kruskal–Wallis test), and some of the medium-high concentrations of nitrate at these sites is likely to have been re-generated by nitrifiers [19]. Regardless, our data indicate ammonia-oxidizers were present and transcriptionally active under all conditions, including dysoxic and pristine (low N and DOC, site C). AOA and AOB can be active at DO concentrations <1 mg/L [46] comparable to, or below, those at the dysoxic site in this study, and as may be found in oxygen-depleted niches within aquifer biofilms [47]. Although, under oxygen depleted conditions such as these, AOB could instead undertake nitrite-dependent ammonia oxidation (‘nitrifier denitrification’), generating N_2_O [48, 49].

Among non-assimilatory pathways, ammonia-oxidation prevailed in pristine groundwater. Site C contained significantly more *nasA* genes, which comprises part of the assimilatory nitrate reduction pathway (*p* < 0.05), along with genes involved in amino acid biosynthesis such as asparagine synthetase (*asnB*) and L-asparaginase II (*ansB*) [50]. This suggests site C had enhanced potential for N uptake and storage, likely due to the large investment required to scavenge nitrogen for cellular maintenance with low nitrate and ammonia concentrations [51].

#### Nitrogen cycling is a core function of groundwater microbiomes, but community compositions are site-specific

**Fig. 3:**
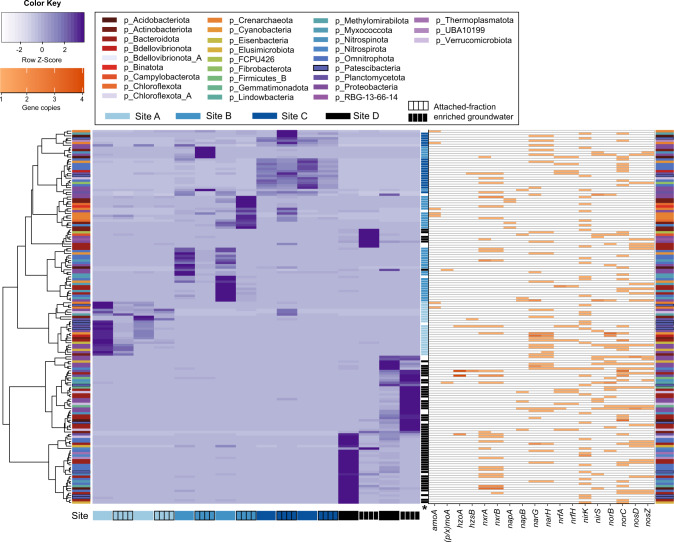
Heatmap showing 159 MAGs, coloured according to phylum, that contain nitrogen-cycling genes involved in non-assimilatory reduction and oxidation of N species in groundwater. Purple gradient (right) represents genome coverage scaled by row (*Z*-Score) across sites A–D (ordered gwj01-16). Rows = MAGs; columns = samples per site (groundwater and attached-fraction enriched groundwater). Orange gradient (right) represents number of nitrogen-cycling gene copies per genome. Microorganisms are ordered based on hierarchical clustering of abundance based Bray-Curtis dissimilarity matrix with ward.D2 clustering method. Final column (labelled with asterisk) indicates genomes that were significantly more abundant at a particular site (coloured rectangle) based on LEfSe analysis.

**Fig. 4:**
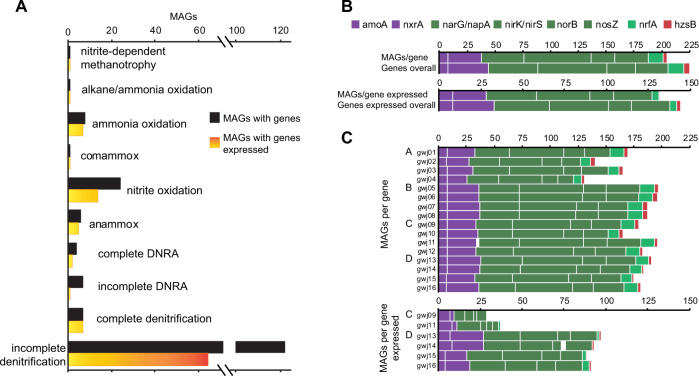
Plots showing the distribution of N cycling mechanisms across MAGs, including DNRA and denitrification pathway fragmentation. **A** Number of MAGs with genes or genes expressed per pathway, with indicative genes required for each step from nitrate to ammonia or N_2_ to denote complete DNRA or denitrification potential, respectively. **B** Number of MAGs with marker genes and marker genes overall for key N cycling processes: two steps for nitrification (purple), four steps for denitrification (dark green), DNRA (light green), and anammox (red). **C** Number of MAGs with at least one copy of each marker gene or marker gene expressed across groundwater samples and sites (A–D).

**Fig. 5:**
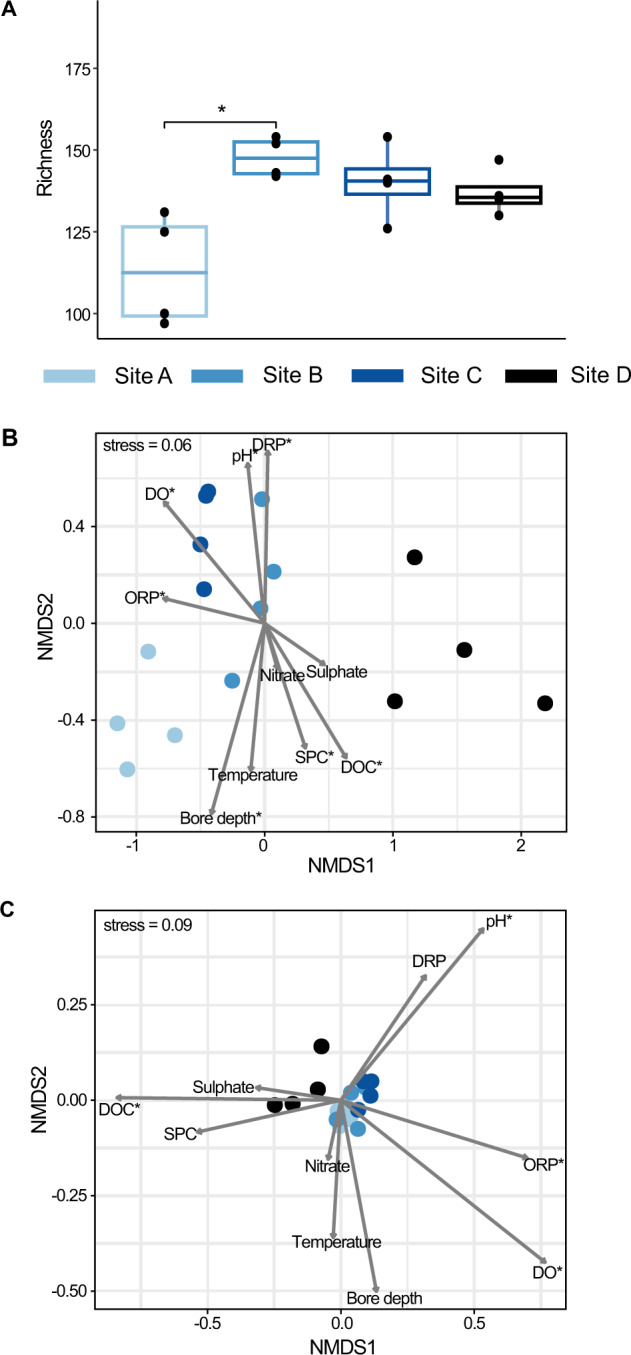
Diversity of community fractions with N cycling capacity. **A** Boxplots showing the median and interquartile range of richness of MAGs capable of nitrogen-cycling. Black solid circles show actual sample richness. Sites with significantly different richness are indicated by an overlying line and asterisk (Wilcoxon test, *p* < 0.05). **B** Non-metric Multi-dimensional Scaling plot showing groundwater community relatedness based on a Bray–Curtis dissimilarity matrix constructed using the relative abundance of MAGs with nitrogen-cycling genes. **C** Non-metric Multi-dimensional Scaling plot based on a Bray–Curtis dissimilarity matrix constructed using the relative abundance of protein-coding sequences in metagenomic reads. For ordinations environmental variables (Table S1) were fitted using envfit, and significant variables are indicated with an asterisk = *p* < 0.05. Ammoniacal-N and nitrite are not shown as >50% of values were below the detection limit and >50% of TKN values are missing.

To link pathways to organisms we analyzed 396 non-redundant medium-high to high-quality MAGs [52]. We reconstructed 7695 metagenome-assembled genomes (MAGs) of which 626 were non-redundant (ANI threshold of 99%), and 396 of these were estimated to be 70–100% complete, with 0–5% contamination. Nitrogen-cycling genes, specifically those involved in non-assimilatory redox reactions, such as nitrification, anammox, and complete or incomplete DNRA and denitrification (starting from nitrate reduction), were present in 40% of MAGs (Figs. 3, 4A, and Table S7). The capacity for diverse nitrogen-cycling processes was again observed to be pervasive across sites, and the overall richness of taxa capable of nitrogen cycling remained comparably diverse over most sites (Fig. 5A), regardless of large differences in measured inorganic N contents (>10-fold difference in nitrate-N, site averages 0–0.007 gm^3^ nitrite-N and 0–0.018 g/m^3^ ammoniacal-N). Analysis of 16S rRNA gene amplicons across 59 groundwater wells likewise shows that taxa linked through phylogenetic affiliation to nitrogen-cycling processes comprise a notable fraction (0.3–26.3%) of complex oxic to anoxic groundwater communities (Fig. S2). Functional redundancy was common among N-cycling microorganisms. Multiple MAGs recovered from each sample had the collective capacity for DNRA and actively expressed genes associated with each of the major steps of nitrification and denitrification (Fig. 4B, C).

Results suggest that nitrogen cycling is a core function of aquifer microbiomes, despite typically low levels of inorganic N in groundwater [4]. This conjecture is supported by prior evidence from ammonium- or nitrate-poor groundwater of microorganisms capable of, or actively engaged in, nitrification, anammox, or denitrification [25, 26, 28, 53, 54]. Microbial nitrogen cycling is likely to be a significant factor governing nitrogen availability in typically oligotrophic habitats, such as groundwater, the open ocean, and lakes. Indeed, in oligotrophic ocean waters with low primary production, the turnover of the dissolved inorganic nitrogen pool via microbial ammonium regeneration and nitrification is rapid [55]. Moreover, numerous microorganisms have evolved high affinities for nitrogen compounds, conferring them with competitive advantages under N-limited conditions [47, 56–59].

Analysis of the spatial distributions of MAGs showed distinct site-specific compositions of bacteria and archaea capable of nitrogen cycling (Fig. 3), a feature also observed in groundwater microbial communities as a whole [13]. Bray–Curtis dissimilarities, calculated based on relative abundance for MAGs and metagenomic reads encoding nitrogen-cycling proteins, revealed that spatial differences significantly influenced community composition (*R*^2^ = 0.49 for MAGs, *R*^2^ = 0.42 for metagenomic reads), more than DO, DOC, nitrate, and sample type (groundwater or attached-fraction enriched) (Fig. 5A, B; Table S8). Most MAGs inferred to undertake nitrogen cycling (87.4%) were significantly more abundant at a specific site (22–57 MAGs/site; LEfSe, Kruskal–Wallis test). Results therefore show site-specific environmental conditions drive species selection, and the capacity for certain reactions, such as ammonia oxidation (greater in oxic groundwater) or complete denitrification (greater in dysoxic groundwater) (Fig. S3).

### Nitrifier diversity and activity in groundwater, and habitat specificity

#### Archaeal and bacterial ammonia-oxidizers exhibited distinct niche preferences, but higher similarity in transcriptional activity

**Fig. 6:**
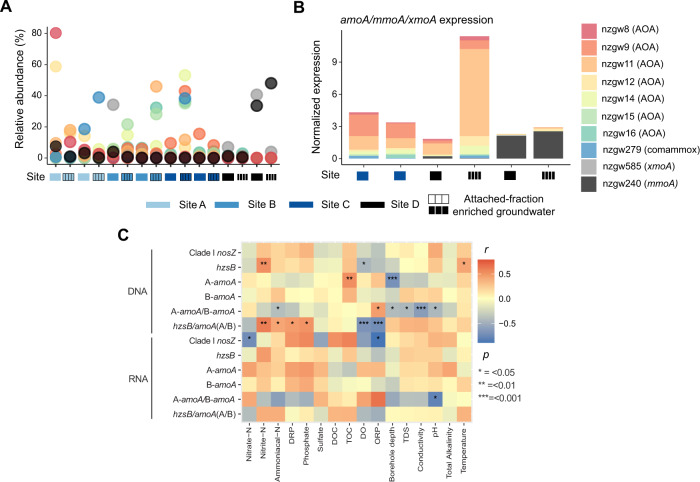
Composition and transcriptional activity of ammonia-oxidizers, and relationship between ammonia-oxidizers, denitrifiers and anammox bacteria to geochemical and physical parameters. **A** Relative abundance of each MAG capable of aerobic ammonia oxidation across the sites. **B** Stacked barplot showing modified transcripts per million, normalized to genome coverage, of *amoA*, *pmoA* and *xmoA* across sites (wells SR1, SR2, E1, N3). The 7 AOA are associated with 5 genera: *Nitrosotenuis* (nzgw11), UBA8516 (nzgw12–14), *Nitrososphaera* (nzgw16), *Nitrosoarchaeum* (nzgw8-9) and an unclassified genus in *Nitrososphaeraceae* (nzgw15). The *xmoA* is part of a gene cluster in MAG nzgw585, recovered from the dysoxic site and classified as *Gammaproteobacteria* genus *Nevskia*, which encodes a copper-containing membrane monooxygenase (CuMMO/*xmoCAB*). The alpha subunit had best hits to *Polycyclovorans* sp. SAT60 and gammaproteobacterial isolate MMS_B.mb.28 (85.71% amino acid identity, NCBI NR database). CuMMO catalyzes the oxidation of short-chain alkanes, ammonia or methane [103]. **C** Spearman’s rank correlations between the abundance (copies/L) of nitrogen-cycling genes (DNA, samples = 64) and transcripts (RNA, samples = 26 above detection limit) determined via ddPCR and geochemical parameters (Table S11). Significant correlations are indicated by * are based on Bonferroni adjusted *p* values (*p*).

Ammonia-oxidizing archaea (AOA) and bacteria (AOB) convert ammonia to nitrite using ammonia monooxygenase (Amo) and hydroxylamine oxidoreductase (Hao), and perform the rate-limiting step in nitrification [60]. Niche differentiation between the two domains is not clearly defined [57]. However, AOA usually have a higher affinity for ammonia than AOB [47, 49, 56], and typically outnumber AOB in oligotrophic environments with low ammonia concentrations and salinity, consistent with groundwater in this study (mean ammoniacal-N 0.36 g/m^3^ ± 1.4 SD; conductivity 220 µS/cm ± 142 SD). We found AOA and AOB exhibited distinct spatial trends in relative and absolute abundance, and activity related to various geochemical parameters in groundwater (e.g. ammonia, oxygen availability, and conductivity) (Fig. 6).

Based on metagenomic reads and MAGs, protein-coding sequences for ammonia monooxygenase (Amo) subunits were most abundant at oxic site A (Figs. 1d, 6A; LEfSe, Kruskal–Wallis test). This trend was mostly driven by AOA, as there were significantly more sequences encoding AOA-AmoA than AOB-AmoA overall at oxic sites (Wilcoxon rank, *p* < 0.05), presumably due to lower ammonia regeneration (measured ammonia/ammonium concentrations were low across all sites regardless of oxygen content, >1 gm^−3^ in only 6% of wells, and most below detection, Table S1). While not all ammonia oxidizer diversity may have been captured by the ddPCR primer sets used (e.g. the AOA primer set has a known bias against some ammonia-oxidizing *Thermoproteota*/*Thaumarchaeota*) [61], quantification of *amoA* genes and transcripts demonstrated a similar relationship with oxygen across a wider set of groundwater sites (Fig. 6C). Archaeal/bacterial *amoA* gene ratios were significantly and positively correlated with ORP (Spearman’s *r* = 0.39), and negatively correlated with borehole depth (Spearman’s *r* = −0.35; Table S9), which is expected to become increasingly oxygen-depleted with depth [62]. These gene ratios were also significantly and negatively associated with ammonia concentrations, conductivity, TDS, and pH (Spearman’s *r* = −0.34 to 52). Transcript ratios exhibited similar trends, albeit significant only for pH.

Taken together, results indicate that AOA and AOB abundance and activity are governed by distinct environmental niches in groundwater, as found in soils [63]. However, while AOA *amoA* gene concentrations were on average 40x higher than AOB genes, this difference was ten-fold less for transcripts (Table S10). Moreover, the deficit in significant correlations between AOA/AOB *amoA* transcript ratios and geochemical/physical groundwater parameters (Fig. 6), suggests comparatively little difference in AOA and AOB activity overall.

#### Ammonia-oxidizers constituted several major lineages, including a single comammox bacterium

Commensurate with a greater abundance of AOA, we reconstructed seven AOA MAGs, along with one *Nitrospiraceae* MAG capable of complete ammonia oxidation (comammox). All contained at least one ammonia monooxygenase gene and six contained genes encoding all AmoABC subunits (Table S7). AOA genomes (and their *amoA* genes, Figs. S4-S6) were phylogenetically diverse, with the MAGs comprising five different genera (Fig. 6a). Of these, nzgw14 (UBA8516) was the most abundant nitrogen cycling MAG overall (Fig. S7), and was most abundant at oxic sites, along with other AOA MAGs. However, AOA MAG relative abundances did not reflect their transcriptional activity (Fig. 6B).

Recently characterized comammox bacteria (*Nitrospira*, phylum *Nitrospirae*) [64] oxidize ammonia to nitrate in three steps (ammonia → hydroxylamine → nitrite → nitrate). *Nitrospiraceae* MAG (nzgw279) possesses genes for ammonia oxidation (*amoABC*), hydroxylamine oxidation (*haoAB*), and nitrite oxidoreductase (*nxrAB*), consistent with comammox. It also possesses a dissimilatory nitrite reductase (*nirK*) present in comammox bacteria elsewhere [65, 66]. Based on 120 concatenated bacterial marker genes (GTDB-Tk) and the AmoA subunit, nzgw279 is closely related to clade B sublineage II comammox *Nitrospira*, and is most similar to *Nitrospira* sp. RCB obtained from an aquifer in Colorado (USA) [66] (Fig. S5), indicating strong habitat driven selection, independent of geographical distance. As expected for comammox, nzgw279 relative abundance was positively correlated with ORP and DO (Spearman’s *r* = 0.87 and 0.63; respectively). Although comammox bacteria can be the most abundant ammonia-oxidizers in some settings (e.g. groundwater-fed sand filters, forest soils and biofilters [67–69]), AOA genomes were more abundant overall here (Figs. 6, S7). Nevertheless, nzgw279 was highly active in terms of *nx*r (although not *amoA*) gene expression (Fig. 2C).

#### Diverse taxa with nitrite oxidoreductase homologues, including Nitrososphaerales

In addition to comammox bacterium nzgw279, we recovered the genomes of two *Nitrospiraceae* that we predict are canonical nitrite oxidizers, nzgw274 (no genus designation; *nxrA* gene present), and nzgw276 (genus=40CM-3-62-11; *nxrAB* present) (Table S7). Neither are affiliated with known comammox bacteria (*Nitrospira* spp. [66]). The *nxrA* from nzgw274 was expressed highest at dysoxic site D in attached-fraction enriched groundwater (*nxrA*, planktonic-fraction = 0.36, attached-fraction = 8.97 TPM). MAG nzgw276 transcripts were also present, but at a much lower level. Results suggest that the well (E1) had sufficient oxygen for NOB to exist. NOB can be active at nanomolar-to-micromolar concentrations of DO [70], and thereby compete for nitrite alongside anammox and denitrifying bacteria.

Known NXR are reported to be genetically diverse [71, 72]. The known diversity of nitrifiers continues to grow [73] and NXR has alternative uses, for example, as a nitrate reductase [71, 72]. Here, homologues were present in a diverse range of other MAGs, including several anammox bacteria (*Planctomycetota*, class *Ca*. Brocadiae, Table S7) [15], which typically use NXR to oxidize a small amount nitrite to nitrate during anammox [74], an archaeal *Nitrososphaerales* (nzgw5; *nxrABC* present), which belongs to a taxonomic group more typically associated with ammonia oxidation (this dataset, Table S7) [74], and various other bacterial phyla, which represent a pool of potentially novel nitrifiers (Fig. S8a).

To further explore NXR in the archaeal *Nitrososphaerales* MAG (nzgw5), we evaluated the 14,591 bp long contig (3135) on which the genes were found. The contig primarily comprises protein-coding genes with closest homology to archaea (based on the NCBI nr database) that are located on either side of a syntenous bacterial-like *nxrABC* gene cluster (Fig. S8b; Table S11), including chaperone-encoding *torD* directly adjacent to *nxrC*, as found in *Nitrospina gracilis*, which is thought to facilitate Mo cofactor maturation and insertion into NxrA [75]. To determine whether *nxr* genes were reproducibly present in other closely related *Nitrososphaerales* genomes, we searched for dereplicated MAGs sharing >99% ANI with nzgw5. We found one “replicate” *Nitrososphaerales* MAG (nzgw5-b) sharing 99.2% ANI, which was recovered from site B (nzgw5 derived from a co-assembly of site B samples), and that possessed a similar *nxrABC* gene cluster (Table S11).

The MAG nzgw5 shares NxrA protein sequence similarity with bacteria as phylogenetically diverse as *Nitrospira defluvii*, *Nitrospina gracilis*, and *Candidatus* Brocadia (Fig. S8a), but the highest NCBI nr database matches were to other aquifer bacteria, Candidate division *Zixibacteria* bacterium RBG-1 (58.56% identity) and *Planctomycetes* bacterium RIFCSPHIGHO2_02_FULL_52_58 (56.7% identity)—both originally recovered genomically from an aquifer in Rifle, CO, USA [11, 76]. NxrB similarly had the highest match with other aquifer-derived genomes (also U.S.A.), including one *Nitrospinae* bacterium (65.97% identity), and most notably, two archaea affiliated with the *Thermoproteota* (previously *Thaumarchaeota*) phylum—another *Nitrososphaerales*, and *Thermoproteota* bacterium (68.71–71.87% identity) [13]. While the gene cluster appears to have been horizontally acquired, we found no identifiable genomic island associated with contig 3135. However, lateral gene transfer occurs more frequently between organisms, including unrelated taxa, that share a habitat [77]. Results suggest aquifer-adapted *Nitrososphaerales* acquired *nxr* genes, and potentially also the ability for nitrite oxidation/reduction, from a co-occurring bacterium.

### Nitrite-dependent methanotroph (*Ca*. Methylomirabilis)

#### Nitrite-dependent methanotrophs were prevalent

Analysis of metagenomic reads revealed that copper-containing, membrane-associated particulate methane monooxygenase subunit A (PmoA) sequences (a closely related AmoA homologue [78]) were ubiquitous, but significantly more abundant at oxic site A (Fig. 1C). This protein is associated with aerobic and anaerobic nitrite-dependent methanotrophs that are able to oxidize methane to CO_2_, using methane as a sole carbon and energy source [79]. Methanotrophic bacteria can also oxidize ammonia to nitrite using particulate methane monooxygenase (pMMO) and a unique hydroxylamine oxidoreductase, HAO [80]. Interactions between methanotrophs and ammonia-oxidizers in aquifers are poorly understood. They are associated with opposite gradients of ammonia and methane, as ammonia inhibits the activity of methanotrophs and methane acts as a competitive inhibitor for ammonia oxidizers [81, 82]. Analysis of 16S rRNA gene amplicons across a wide distribution of aquifer samples (*n* = 80) showed that *Ca*. Methylomirabilis relative abundance was positively correlated with ammonia-oxidizers *Nitrosotaleacae, Nitrosomonadaceae*, and *Nitrosopumilaceae* (Spearman’s *r* = 0.42, *r* = 0.33 and *r* = 0.37, respectively). Aerobic ammonia-oxidizers consume O_2_ and may provide a habitable environment for *Ca*. Methylomirabilis at oxic/anoxic interfaces and produce nitrite, which could potentially be directly used by these nitrite-dependent methanotrophs [83].

We recovered one nitrogen cycling methanotrophic MAG (nzgw240), related to the anaerobic methane-oxidizing genus *Ca*. Methylomirabilis (Figs. 6, S6) [84]. Members of *Ca*. Methylomirabilis perform NO_2_^−^ dependent anaerobic methane oxidation through an intra‐aerobic pathway involving the dismutation of NO into O_2_ and N_2_ [79, 84], and play an important role in controlling N_2_O and methane emissions from natural ecosystems [79]. All *pmoABC* subunits for methane oxidation [79] were present in nzgw240, and were closely related to those from *Ca*. Methylomirabilis lanthanidiphila (74.80–95.5% amino acid identity, Fig S6), a methanotroph that dominated an enrichment culture after addition of rare-earth metal cerium [85]. Several genes involved in nitrogen cycling, such as nitrate oxidoreductase (*nxrAB*), nitrite reductase (*nirS*), putative NO dismutase (*nod*) and nitric oxide reductase (*norZ)* were also identified in MAG nzgw240, and were expressed at the dysoxic site alongside *pmoA* (Figs. 2c, 6b), where dissolved methane was also detected [86]. The first of two nitric oxide-like reductases shares 95.6% amino acid identity with Nod (DAMO_2434) in *Ca*. Methylomirabilis oxyfera [87]. This enyzme is homologous to the quinol-dependent NO reductases (qNOR) [87], however experimental validation is still required to prove nitric oxide disproportionation. The second is a NO reductase sharing 88.34% amino acid identity with NorZ (DAMO_1889).

### Final-step of denitrification

#### Atypical NosZ was more common than typical NosZ

**Fig. 7:**
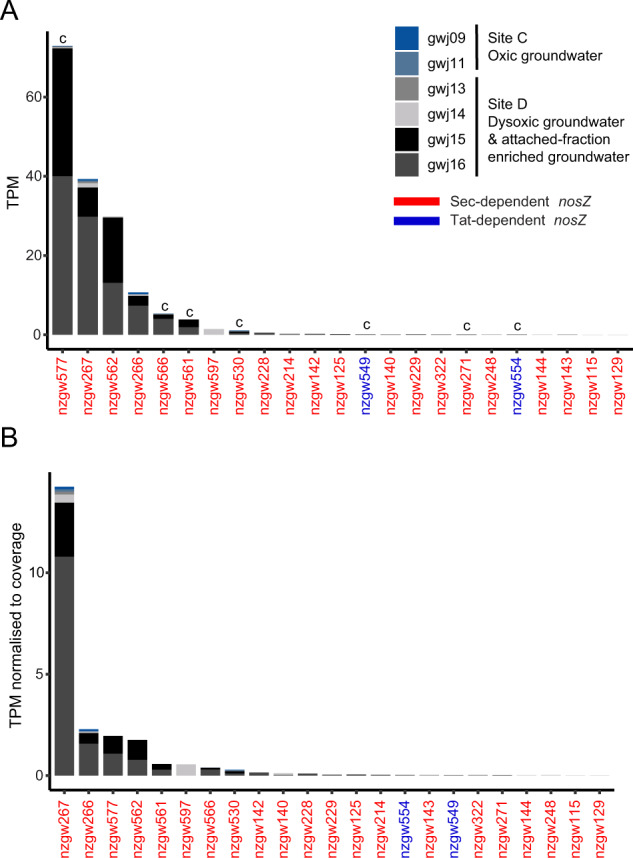
Nitrous-oxide reductase (*nosZ*) gene transcripts in sites C and D groundwater and attached-fraction enriched groundwater. **A** Stacked barplot shows modified-TPM of Sec- and Tat- dependent *nosZ* genes at each site. **B** Stacked barplot shows modified-TPM normalized to genome coverage of Sec- and Tat-dependent *nosZ* genes at each site. While complete denitrifier, *Sulfuricella* MAG nzgw577, contributed the most transcripts, after normalizing to MAG relative abundance, *nosZ* genes from two novel *Nitrospinota* MAGs (nzgw266-267, class UBA7883 [104]) were transcriptionally most active. c = MAGs which contain genes for the complete denitrification pathway (nzgw numbers: 271, 530, 549, 554, 561, 566, and 577).

Thirty MAGs, spanning 10 bacterial phyla, contained *nosZ* genes (Fig. 3). The NosZ protein catalyzes the conversion of green-house gas N_2_O to N_2_ in the last step of denitrification. Typical NosZ proteins (clade I) contain a twin-arginine translocation (Tat) signal peptide, and to date are affiliated exclusively with *Proteobacteria*, which usually perform complete denitrification [88]. A maximum-likelihood tree revealed that NosZ predicted protein sequences comprised both typical clade I twin-arginine (Tat) dependant N_2_O reductase with Proteobacteria (2 MAGs) and atypical clade II secretory (Sec) dependent N_2_O reductase proteins (23 MAGs) (Fig. S9) [88]. The tree also shows a novel clade of *Nitrospirota* and *Nitrospinae* NosZ Sec-dependant sequences, including four NosZ sequences from this study (Fig. S9) that were transcriptionally active (Fig. 7). Members of clade II are considered non-denitrifiers, typically performing just the final step of denitrification [89]. However, 4/7 MAGs capable of complete denitrification encode clade II NosZ, demonstrating complete denitrifiers are present across both clades. Sec-dependent protein translocation is considered more energetically favourable than Tat, requiring between 700 and 5,000 molecules (or equivalent) of ATP per protein translocation across the membrane, whereas Tat requires the equivalent of ~10,000 molecules of ATP [90]. A greater proportion of Sec signal pathways in low nutrient groundwater would be favourable for energy conservation.

#### Nitrous oxide reductase gene expression was strongly associated with oxygen availability and not limited by pathway fragmentation

Based on ddPCR, *nosZ* clade I genes (3 × 10^1^–6 × 10^5^ copies/L) and transcripts (1 × 10^1^–7 × 10^4^ copies/L) were detected across most aquifer samples tested (Table S10). Expression was significantly and negatively correlated with ORP (Fig. 6), reflecting observations elsewhere that the rate of denitrification decreases linearly with increasing ORP [91]. Accordingly, at the dysoxic site there was also a proportionally higher abundance of MAGs with *nosZ* genes of any type (Fig. 3), and of complete denitrifier MAGs, which comprised up to 42% of the nitrogen cycling community (4–31× more on average than oxic sites) (Fig. S3).

N_2_O generation due to incomplete denitrification has been shown to be highest under oxic conditions in groundwater [92], comparable to sites A-C here. A fragmented denitrification pathway may explain higher N_2_O concentrations in some oxic groundwaters. Fragmented genetic potential for biogeochemical cycling processes, such as denitrification, appears to be a common trait in aquifer bacteria (Fig. 4) [11], necessitating metabolic handoffs among individuals to complete pathways. Our transcriptomic data points to active collaboration among incomplete denitrifiers for generation and removal of N_2_O (although in situ measurements would be required to determine the presence or absence of NO or N_2_O emissions). Transcriptional activity associated with N_2_O reduction was at least equivalent to that for generation, regardless of groundwater oxygen-content or the portion of pathway fragmentation (Fig. 2A).

### Transcriptional activity of co-occurring aerobic and anaerobic nitrogen-cycling pathways in oxic versus dysoxic groundwater

Based on transcripts mapped to MAGs, there was less nitrogen-cycling transcriptional activity at the pristine oxic site compared to the dysoxic site (average modified-TPM 69 ± 13 site C versus 359 ± 362 site D) (Fig. 2B). This is consistent with quantification of 8x more nitrogen-cycling transcripts by ddPCR in dysoxic versus oxic groundwater, across a wider set of groundwaters (on average 7 × 10^4^ transcripts/L in dysoxic and 8 × 10^3^ transcripts/L in oxic groundwater; Table S11). The greatest proportion of mapped transcripts at the oxic site was associated with ammonia oxidation to nitrate and re-reduction to nitrite, based on ammonia monooxygenase (*amo*), nitrite oxidoreductase (*nxr*), and periplasmic nitrate reductase (*nap*) gene transcripts (Fig. 2A). Expression of *nap* genes is suggestive of aerobic denitrification at this site, as nitrate reduction in the periplasm is not inhibited by oxygen [93].

At the dysoxic site, the greatest portion of gene expression in groundwater and attached-fraction enriched groundwater, based on mapped transcripts was associated with, again, nitrite oxidation (*nxr*), but also anammox (*hzo*, *hzs*), and denitrification (*nor* and *nos*) (Fig. 2A). These genes each contributed up to 8−66% of nitrogen-cycling transcripts at site D. Hydrazine synthase *hzsB* transcripts (quantified by ddPCR) were also highest at the dysoxic site (April 2018), one of only seven sites in the study with detectable nitrite concentrations. Contemporaneous measurements of excess N_2_ indicated active N_2_ generation in dysoxic groundwater from this site (wells E1 and N3; Table S12) due denitrification and/or anammox [15]—the technique cannot distinguish between N_2_ produced by these processes. Nitrate-based δ^18^O against δ^15^N measurements from groundwater in well N3 also indicated the occurrence of denitrification [86]. The majority of *hzoA and hzsB* genes were expressed by just two *Planctomycetes* MAGs (nzgw511–512) at this site (37% and 58%, respectively). However, when considering *hzsB* transcript concentrations in the wider aquifer dataset, we observed no relationship with DOC or oxygen availability (DO, ORP or borehole depth) (Fig. 6), indicating these bacteria are active under a wide variety of groundwater conditions, including those considered unfavourable for anammox (i.e. high DOC and DO). *Ca*. Brocadiae genomes recovered from Sites A–D were previously found to have a broad range of ABC transport systems and variations in substrate importation such as phosphate, cobalt, nickel, iron(III), zinc, sulfate, molybdate, lipoproteins, ribose, rhamnose, polysaccharides, and oligopeptides, suggesting that they may not just be autotrophic specialists [15]. We found evidence for greater competition among N_2_O reducers (Fig. 2B), although most *nosZ* transcripts overall (81%) were expressed by a single complete denitrifier, MAG nzgw577 (genus *Sulfuricella*; Fig. 7) at the dysoxic site (Fig. 2C), despite it being only the third most active in terms of *nosZ* expression after normalizing to MAG coverage (Fig. 7). Members of this genus are reported to perform autotrophic denitrification coupled with the oxidation of reduced sulfur compounds [94].

Several aerobic ammonia-oxidizers, for which we recovered MAGs, were also active at the dysoxic site, contributing 5-6% of nitrogen-cycling transcripts mapped (Fig. 2B). This suggests the potential for simultaneous nitrification and denitrification, and partial coupling of these pathways (overall modified-TPM 1:2.7 *amoA*:*nosZ*, 1:2.3 *nosZ*:*nxrA*). Most (88%) *amoA* transcriptional activity at this dysoxic site was attributed to four AOA MAGs affiliated with *Nitrosopumilaceae*. The greatest proportion of ammonia monooxygenase transcription was associated with the attached-fraction enriched groundwater from this site (Fig. 6B), consistent with preferences previously reported for the ammonia-oxidizing genera, *Nitrososphaera* and *Nitrosopumilus*, in groundwater [30]. Prior findings from karstic aquifers also suggest that soil-derived ammonia oxidizers may be imported into groundwater [26], which may be important for shallow aquifers that directly receive leachate from the soil zone, along with any microorganisms it carries.

The most transcribed nitrogen-cycling gene among all MAGs was *nxrB* from comammox *Nitrospiraceae* nzgw279, which showed the highest expression in attached-fraction enriched groundwater at site D. This groundwater contained more total suspended solids (Table S1), and therefore more sediment particles coated in biofilms [95]. Comammox *Nitrospira* populations have previously been found to dominate biofilms in wastewater, outnumbering all other nitrifiers [96]. As nzgw279 was associated with fewer *amoA* transcripts than other ammonia-oxidizers (average modified-TPM 0.77 ± 1.02 SD vs 2.01 ± 3.22 SD), and appeared to act largely as a canonical nitrifier [97], it potentially received nitrite as a by-product from the several active AOAs.

Results show methanotroph methane monooxygenase gene transcription occurred alongside gene expression associated with nitrification, anammox, and denitrification at the dysoxic site. Methanotrophs and ammonia-oxidizers share many metabolic similarities based on a common evolutionary history, and supported by the structural similarities of ammonia and methane monooxygenases [81], methanotrophs have been implicated in both methane and ammonia oxidation in groundwater [98]. In this study, *Ca*. Methylomirabilis (nzgw240) expressed genes, associated with concurrent methane oxidation (*pmoA*) and nitric oxide dismutation (NO_2_^-^ reductase *nirS* and NO dismutase *nod*) to N_2_ and O_2_ (Fig. 2C) [84]. Co-occurring gene expression reveals a potential interaction among AOA, *Ca*. Methylomirabilis, and anammox bacteria, whereby nitrite produced from aerobic ammonia oxidation by AOA, drives anammox and nitrite-dependent anaerobic methane oxidation by *Ca*. Methylomirabilis*. Ca*. Methylomirabilis produces oxygen which could create an interface whereby AOA and comammox can co-exist. Oxygen consumption and nitrite provisioning by AOA could represent synergism with anammox in the terrestrial subsurface, as previously predicted to occur in unconfined aquifer soils [99]. Indeed, ammonia oxidizer and anammox activities, based on transcript copy numbers, were found to be tightly linked across distinct groundwater chemistries in the wider set of samples [15]. These heterogeneous reactions at the dysoxic site indicate that it likely contained mixed redox conditions in situ. This could be due to oxygen penetration from above [100], and vertical stratification of electron donors [101], geochemical gradients created by biofilm formation [102], or oxygen produced by the intra-aerobic pathway of *Ca*. Methylomirabilis species [84].

## Conclusion

Results show that the capacity for non-assimilatory nitrogen-cycling reactions, such as ammonia oxidation and denitrification, was prevalent in groundwater regardless of site-specific physicochemistry, although the relative abundance of each pathway differed. Phylogenetically diverse AOA and AOB were associated with distinct environmental niches in groundwater, and AOA-*amoA* genes and transcripts were more abundant overall. While incomplete denitrifiers were numerous, complete denitrifiers contributed to a substantial fraction of transcriptional activity under dysoxic conditions, where activity associated with denitrification, and N-cycling transcripts was greatest. Gene expression associated with nitrification, denitrification, nitrite-dependent methane oxidation, and anammox occurred simultaneously in dysoxic groundwater, such that nitrite (or nitrate) produced by AOA or comammox could fuel anammox, denitrification, and methanotrophy by *Ca*. Methylomirabilis. Results provide insights into microbial N-transformations in groundwater with distinct chemical characteristics (such as oxygen availability and DOC), and potential metabolic “handoffs” among nitrogen-cycling organisms.

## Supplementary information

Supplementary materials

Dataset 1

## Data Availability

Sequences are deposited with NCBI under BioProject PRJNA699054.
