# Supplementary material for: Nitrogen cycling and microbial cooperation in the terrestrial subsurface
Source: ISME J. 2022 Aug 8;16(11):2561–73. doi: 10.1038/s41396-022-01300-0 (PMC9562985; doi:10.1038/s41396-022-01300-0)
Supplement: Supplementary file 1 — Supplementary materials [file 41396_2022_1300_MOESM1_ESM.docx]

**SUPPLEMENTARY INFORMATION**

**SUPPLEMENTARY METHODS**

*Geochemical measurements*

Total phosphorus and phosphate were determined according to American Public Health Association (APHA) 4500-P B & E (modified to include an acidic ammonium persulphate to convert organophosphates and polyphosphates to orthophosphate), measured using a Konelab 60 Discrete Analyser [1]. Dissolved reactive phosphorus (DRP) was determined according to APHA 4500-P G (sample was reacted with ammonium molybdate and ascorbic acid to form molybdenum blue then detected at 880 nm). Total ammoniacal-N was determined according to APHA 4500-NH3 H (using phenol/hypochlorite reaction forming a complex that was detected at 630 nm) and calculated as NH_4_-N = NH_4_^+^-N + NH_3_-N. Nitrite-N and Nitrate-N + Nitrite-N were determined according to APHA 4500-NO_3_ I (NOxN is measured via automated cadmium reduction and griess reaction. NO_2_N calculated by automated griess reaction (Sulfanilamide) detected at 540nm. Nitrate-N was calculated by (Nitrate-N + Nitrite-N) – Nitrite-N. DRP, total ammoniacal-N, nitrite-N and nitrate were measured using a Lachat QC8500 Series 2 Flow Injection Analyser. Total Kjeldahl Nitrogen was determined via digestion and phenol/hypochlorite colorimetry using a discrete Analyser (Konelab 60) according to APHA 4500-Norg D and 4500 NH_3_ F. Total organic carbon (TOC) and dissolved organic carbon (DOC) were measured according to APHA 5310 C (Analysed using Super Critical Persulphate Oxidation with phosphoric acid and sodium persulphate), using a Sievers InnovOx TOC Analyser. TOC = total carbon – total inorganic carbon. For DOC, groundwater was filtered first using 0.45 μm Polypropylene filter (Whatman, Maidstone, UK). Total suspended solids were measured by first evaporating groundwater samples in an oven at 105 °C until dry. Dried solids were then weighed and normalised to the total water volume analysed. Sulfate was measured according to APHA 4110 B. Total copper, and total and dissolved iron were measured by nitric acid digestion using ICP-MS according to APHA 3125 B (to determine dissolved iron, groundwater samples were filtered beforehand). Total dissolved solids were measured by filtering through a 1.2 μm glass fibre filter (Whatman, Maidstone, UK) according to APHA 2540 C 1-5 (modified; drying temperature of 103-105°C used rather than 180 ± 2°C using a Contherm Thermotec2000 Oven). Alkalinity was analysed according to APHA 3125 B.

*Nucleic acid extraction.* Filters were centrifuged (2500 g for 5 min) and washed with nuclease-free Phosphate-Buffered Saline to remove RNAlater. DNA was extracted using DNeasy PowerSoil Pro kits (Qiagen, Valencia, CA, USA) with 0.14–0.89 g of crushed filter per extraction. This represented 0.01-2.91 L of groundwater per sample (x64) for ddPCR (1 extraction/sample; amounts listed in Table S1), and 0.04-12.75 L per sample for metagenomics (x16) and amplicon sequencing (x80) (1-47 extractions/sample), where replicate extractions were pooled and concentrated via ethanol precipitation.

RNA was extracted from aliquots of the same samples using the RNeasy PowerSoil Total RNA kit (Qiagen) with 2.12–3.90 g of crushed filter per extraction. This represented 0.51-7.98 L of groundwater per sample for ddPCR (1 extraction/sample), and 0.06-7.05 L per sample for metatranscriptomics (1-3 extractions/sample). Nuclease-free glycogen was added to aid precipitation (0.1 µg/µL final concentration). RNA was DNase treated using the TURBO DNA-*free*™ Kit (“rigorous” protocol; Invitrogen, Carlsbad, CA, USA). DNA removal was verified via 16S rRNA gene amplification (conditions in Table S2, but over 55 cycles) and gel electrophoresis. Extractions were concentrated using the RNA Clean and Concentrator-5 Kit (Zymo Research, Irvine, CA, USA).

Nucleic acids were quantified with a Qubit 3.0 fluorometer (ThermoFisher Scientific, Waltham, MA, USA) using dsDNA and RNA HS assay kits, and quality checked using a NanoPhotometer (Implen, Munich, Germany). High molecular weight DNA for metagenomics was verified via gel electrophoresis. RNA was further checked using an Agilent BioAnalyzer with RNA 6000 Nano and Pico chips (Santa Clara, CA, USA). Of the 64 samples, 26 samples yielded detectable RNA with RIN ≥6 or DV200 >30% (fragments >200 nucleotides), which were used for downstream analysis.

*Metagenome and metatranscriptome sequencing.* DNA libraries for 15 samples (gwj01–gwj16, sites A-D), were prepared using the TruSeq Nano DNA Kit (Illumina, San Diego, CA, USA), with a targeted fragment size of 550 bp, except low-yield sample gwj02, which was prepared with the ThruPLEX DNA-seq Kit (Takara Bio, Mountain View, CA, USA), at the Otago Genomics Facility (University of Otago, NZ). 2x250 bp paired-end sequencing was performed using the Illumina HiSeq 2500 V4 platform. RNA libraries were prepared using the Ovation SoLo RNA-Seq System (NuGEN, Redwood City, CA, USA), including DNase treatment and custom rRNA depletion probes. Custom rRNA probes were designed by NuGEN using small and large ribosomal subunit sequences reconstructed from the 16 metagenomes with EMIRGE [2] over 40 iterations with clustering at 97% identity and using the SILVA 132 database [3]. Ribosomal sequences generated were used as target sequences in the design of custom AnyDeplete probes using NuGEN’s proprietary algorithm. 2×125 bp paired-end reads were generated using HiSeq 2500 V4.

### *Genome assembly, binning and transcript mapping.* Adapter sequences were removed from reads using Cutadapt v2.10 [4], and trimmed with sickle (Phred score ≥30; read length ≥80 bp) (https://github.com/najoshi/sickle) and quality checked using FastQC v0.11.7 (http://www.bioinformatics.babraham.ac.uk). Residual rRNA sequences were removed from metatranscriptomes using SortMeRNA v2.1 [5], and checked to ensure filtered reads were still paired using BBMap v38.81 “repair.sh” [6]. All metagenomes were individually assembled, and those from the same site (groundwater ± attached-fraction) were also co-assembled, using SPAdes v3.11.1[7] (--meta, -k 43,55,77,99,121). Scaffolds ≥2 kb long were binned with MetaBAT2 v2.12 [8], Maxbin v2.2.6 [9] and CONCOCT v1.0.0 [10]. Best-scoring bins per assembly were selected with DAS_Tool v1.1.1 [11]. Bins were de-replicated across assemblies using dRep v2.0.5 (average nucleotide identity, ANI >99%) [12], and manually refined using *t-SNE* transformation of tetranucleotide frequencies and scaffold coverage values (https://github.com/dwwaite/bin_detangling). Genome completeness and contamination were estimated using CheckM v1.0.12 [13]. For genome coverage, trimmed metagenomics reads were mapped using bowtie v2.3.2 [14] (-n 1 -l 222 --minins 200 --maxins 800 –best), and normalized to library size [15]. Metagenome-assembled genomes (MAGs) were classified using the Genome Taxonomy Database taxonomic classification tool, GTDB-Tk v0.2.1 [16].

Metatranscriptomic reads were mapped to MAGs using Bowtie2 [17] (v2.3.5, --end-to-end --very_sensitive). Read counts were determined using featureCounts [18] (v1.6.3, -F SAF). Singleton mapped reads per gene were removed. Read counts were normalized to a modified version of transcripts per kilobase per million reads mapped (modified-TPM) [19] via: (number of reads mapped to gene)*(1000/gene length)*(1000000/library size).

*Amplicon sequencing and processing*

PCR amplification of 16S rRNA genes used modified Earth Microbiome Project primers EMP-16S-515ʹF and EMP-16S-806ʹR primers [20, 21] with Illumina Nextera adapters, and MyTaq HS Red Mix (Bioline, London, UK). PCR conditions are described in Table S2. Amplicons were purified using Agencourt AMPure XP magnetic beads (Beckman Coulter, Brea CA, USA). Barcoded libraries were prepared by Auckland Genomics (University of Auckland, NZ) according to Illumina's 16S Metagenomic Sequencing Library Preparation manual and were loaded with 10% PhiX, for 2 × 250 bp sequencing via Illumina MiSeq with V2 chemistry.

Sequences were quality checked using FastQC v0.11.7 [22], and merged using USEARCH v9.0.2132 [23]. Sequences were quality filtered using sickle (minimum Phred score ≥30; length ≥200 bp) with another 10 bp of lower quality sequence removed from each end using USEARCH -fastx_truncate [23]. Sequences were dereplicated and clustered at 97% similarity with chimera removal to generate operational taxonomic units (OTUs) using the UCLUST pipeline [24]. OTUs were classified using USEARCH -sintax with SILVA SSU Ref NR99 database v138 [3]. Non-prokaryotic and singleton sequences were removed before rarefying to 13 393 using QIIME2 v2018.2 [25].

*Metabolic predictions*

Protein-coding gene sequences from MAGs and metagenomic reads were predicted using Prodigal v2.6.3 [26]. To characterize nitrogen-cycling gene (sub)families, predicted protein-coding sequences from metagenomic reads were annotated against NCycDB [27] using DIAMOND [28] (e-value=0.001, sampling depth=66,759,303). Predicted protein-coding sequences from MAGs were annotated using USEARCH v9.02132 [23] with -usearch_global command (–id=0.5 –evalue=0.001 –maxhits=10) by searching against the UniRef100 and UniProt [29] (release-2018_10) databases and KEGG (release 86) database [30]. Hidden Markov Model searches were carried out using HMMER v3.3 [31] against Pfam v32 [32] and TIGRfam v14 [33] databases, and against custom databases from Anantharaman et al. [34] (cutoff scores in Table S3). All predicted nitrogen-cycling proteins from MAGs were then checked against the conserved domain database [35], using an 0.001 evalue threshold, and only sequences with the desired conserved domains were kept. Results from all the databases were merged and summarised with a custom python script (“annotationAggregator.py” v0.1, https://github.com/GenomicsAotearoa/environmental_metagenomics).

**REFERENCES**

| 1. | Rice EW, Baird RB, Eaton AD. APHA Standard Methods for the Examination of Water and Wastewater, 23rd Edition 2017. |
| --- | --- |
| 2. | Miller CS, Baker BJ, Thomas BC, Singer SW, Banfield JF. EMIRGE: Reconstruction of full-length ribosomal genes from microbial community short read sequencing data. *Genome Biol* 2011; 12: R44. |
| 3. | Quast C, Pruesse E, Yilmaz P, Gerken J, Schweer T, Yarza P, et al. The SILVA ribosomal RNA gene database project: Improved data processing and web-based tools. *Nucleic Acids Res* 2013; 41: D590–D596. |
| 4. | Martin M. Cutadapt removes adapter sequences from high-throughput sequencing reads. *EMBnet.journal* 2011; 17: 10. |
| 5. | Kopylova E, Noé L, Touzet H. SortMeRNA: Fast and accurate filtering of ribosomal RNAs in metatranscriptomic data. *Bioinformatics* 2012; 28: 3211–3217. |
| 6. | Bushnell B. BBMap: a fast, accurate, splice-aware aligner. Lawrence Berkeley National Laboratory. LBNL-7065E. Retrieved from https://escholarship.org/uc/item/1h3515gn |
| 7. | Bankevich A, Nurk S, Antipov D, Gurevich AA, Dvorkin M, Kulikov AS, et al. SPAdes: A new genome assembly algorithm and its applications to single-cell sequencing. *J Comput Biol* 2012; 19: 455–477. |
| 8. | Kang DD, Li F, Kirton E, Thomas A, Egan R, An H, et al. MetaBAT 2: An adaptive binning algorithm for robust and efficient genome reconstruction from metagenome assemblies. *PeerJ* 2019; 7: e7359. |
| 9. | Wu YW, Tang YH, Tringe SG, Simmons BA, Singer SW. MaxBin: An automated binning method to recover individual genomes from metagenomes using an expectation-maximization algorithm. *Microbiome* 2014; 2: 26. |
| 10. | Alneberg J, Bjarnason BS, De Bruijn I, Schirmer M, Quick J, Ijaz UZ, et al. Binning metagenomic contigs by coverage and composition. *Nat Methods* 2014; 11: 1144–1146. |
| 11. | Sieber CMK, Probst AJ, Sharrar A, Thomas BC, Hess M, Tringe SG, et al. Recovery of genomes from metagenomes via a dereplication, aggregation and scoring strategy. *Nat Microbiol* 2018; 3: 836–843. |
| 12. | Olm MR, Brown CT, Brooks B, Banfield JF. DRep: A tool for fast and accurate genomic comparisons that enables improved genome recovery from metagenomes through de-replication. *ISME J* 2017; 11: 2864–2868. |
| 13. | Parks DH, Imelfort M, Skennerton CT, Hugenholtz P, Tyson GW. CheckM: Assessing the quality of microbial genomes recovered from isolates, single cells, and metagenomes. *Genome Res* 2015; 25: 1043–1055. |
| 14. | Langmead B, Trapnell C, Pop M, Salzberg SL. Ultrafast and memory-efficient alignment of short DNA sequences to the human genome. *Genome Biol* 2009; 10: R25. |
| 15. | Probst AJ, Ladd B, Jarett JK, Geller-McGrath DE, Sieber CMK, Emerson JB, et al. Differential depth distribution of microbial function and putative symbionts through sediment-hosted aquifers in the deep terrestrial subsurface. *Nat Microbiol* 2018; 3: 328–336. |
| 16. | Chaumeil P-A, Mussig AJ, Hugenholtz P, Parks DH. GTDB-Tk: a toolkit to classify genomes with the Genome Taxonomy Database. Bioinformatics. 2019; 36: 1925–1927. |
| 17. | Langmead B, Salzberg SL. Fast gapped-read alignment with Bowtie 2. *Nat Methods* 2012; 9: 357–359. |
| 18. | Liao Y, Smyth GK, Shi W. FeatureCounts: An efficient general purpose program for assigning sequence reads to genomic features. *Bioinformatics* 2014; 30: 923–930. |
| 19. | Wagner GP, Kin K, Lynch VJ. Measurement of mRNA abundance using RNA-seq data: RPKM measure is inconsistent among samples. *Theory Biosci* 2012; 131: 281–285. |
| 20. | Hugerth LW, Wefer HA, Lundin S, Jakobsson HE, Lindberg M, Rodin S, et al. DegePrime, a program for degenerate primer design for broad-taxonomic-range PCR in microbial ecology studies. *Appl Environ Microbiol* 2014; 80: 5116–5123. |
| 21. | Apprill A, Mcnally S, Parsons R, Weber L. Minor revision to V4 region SSU rRNA 806R gene primer greatly increases detection of SAR11 bacterioplankton. *Aquat Microb Ecol* 2015; 75: 129–137. |
| 22. | Andrews S, Krueger F, Segonds-Pichon A, Biggins L, Krueger C, Wingett S. FastQC: a quality control tool for high throughput sequence data. Available online at: http://www.bioinformatics.babraham.ac.uk/projects/fastqc. *Babraham Institute*. |
| 23. | Edgar RC. Search and clustering orders of magnitude faster than BLAST. *Bioinformatics* 2010; 26: 2460–2461. |
| 24. | Edgar RC. UPARSE: Highly accurate OTU sequences from microbial amplicon reads. *Nat Methods* 2013; 10: 996–998. |
| 25. | Caporaso JG, Kuczynski J, Stombaugh J, Bittinger K, Bushman FD, Costello EK, et al. QIIME allows analysis of high-throughput community sequencing data. *Nat Methods* 2010; 7: 335–336. |
| 26. | Hyatt D, Chen GL, LoCascio PF, Land ML, Larimer FW, Hauser LJ. Prodigal: Prokaryotic gene recognition and translation initiation site identification. *BMC Bioinformatics* 2010; 11: 119. |
| 27. | Tu Q, Lin L, Cheng L, Deng Y, He Z. NCycDB: A curated integrative database for fast and accurate metagenomic profiling of nitrogen cycling genes. *Bioinformatics* 2019; 35: 1040–1048. |
| 28. | Buchfink B, Xie C, Huson DH. Fast and sensitive protein alignment using DIAMOND. *Nat Methods* 2014; 12: 59–60. |
| 29. | The UniProt Consortium. UniProt: The universal protein knowledgebase. *Nucleic Acids Res* 2018; 46: D158–D169. |
| 30. | Ogata H, Goto S, Sato K, Fujibuchi W, Bono H, Kanehisa M. KEGG: Kyoto Encyclopedia of Genes and Genomes. *Nucleic Acids Research* 1999; 27: 29–34. |
| 31. | Potter SC, Luciani A, Eddy SR, Park Y, Lopez R, Finn RD. HMMER web server: 2018 update. *Nucleic Acids Res* 2018; 46: W200–W204. |
| 32. | Finn RD, Bateman A, Clements J, Coggill P, Eberhardt RY, Eddy SR, et al. Pfam: The protein families database. *Nucleic Acids Res* 2014; 42: D222–D230. |
| 33. | Haft DH, Selengut JD, White O. The TIGRFAMs database of protein families. *Nucleic Acids Res* 2003; 31: 371–373. |
| 34. | Anantharaman K, Brown CT, Hug LA, Sharon I, Castelle CJ, Probst AJ, et al. Thousands of microbial genomes shed light on interconnected biogeochemical processes in an aquifer system. *Nat Commun* 2016; 7: 1–11. |
| 35. | Lu S, Wang J, Chitsaz F, Derbyshire MK, Geer RC, Gonzales NR, et al. CDD/SPARCLE: The conserved domain database in 2020. *Nucleic Acids Res* 2020; 48: D265–D268. |
| 36. | Schmidt CM, Fisher AT, Racz AJ, Lockwood BS, Huertos ML. Linking denitrification and infiltration rates during managed groundwater recharge. *Environ Sci Technol* 2011; 45: 9634–9640. |
| 37. | Diamond S, Lavy A, Crits-Christoph A, Matheus Carnevali PB, Sharrar A, Williams KH, et al. Soils and sediments host Thermoplasmata archaea encoding novel copper membrane monooxygenases (CuMMOs). *ISME J* 2022; 1–15 https://doi.org/10.1038/s41396-021-01177-5. |
| 38. | Edgar RC. MUSCLE: A multiple sequence alignment method with reduced time and space complexity. *BMC Bioinformatics* 2004; 5: 113. |
| 39. | Capella-Gutiérrez S, Silla-Martínez JM, Gabaldón T. trimAl: A tool for automated alignment trimming in large-scale phylogenetic analyses. *Bioinformatics* 2009; 25: 1972–1973. |
|  |  |

**SUPPLEMENTARY FIGURES**


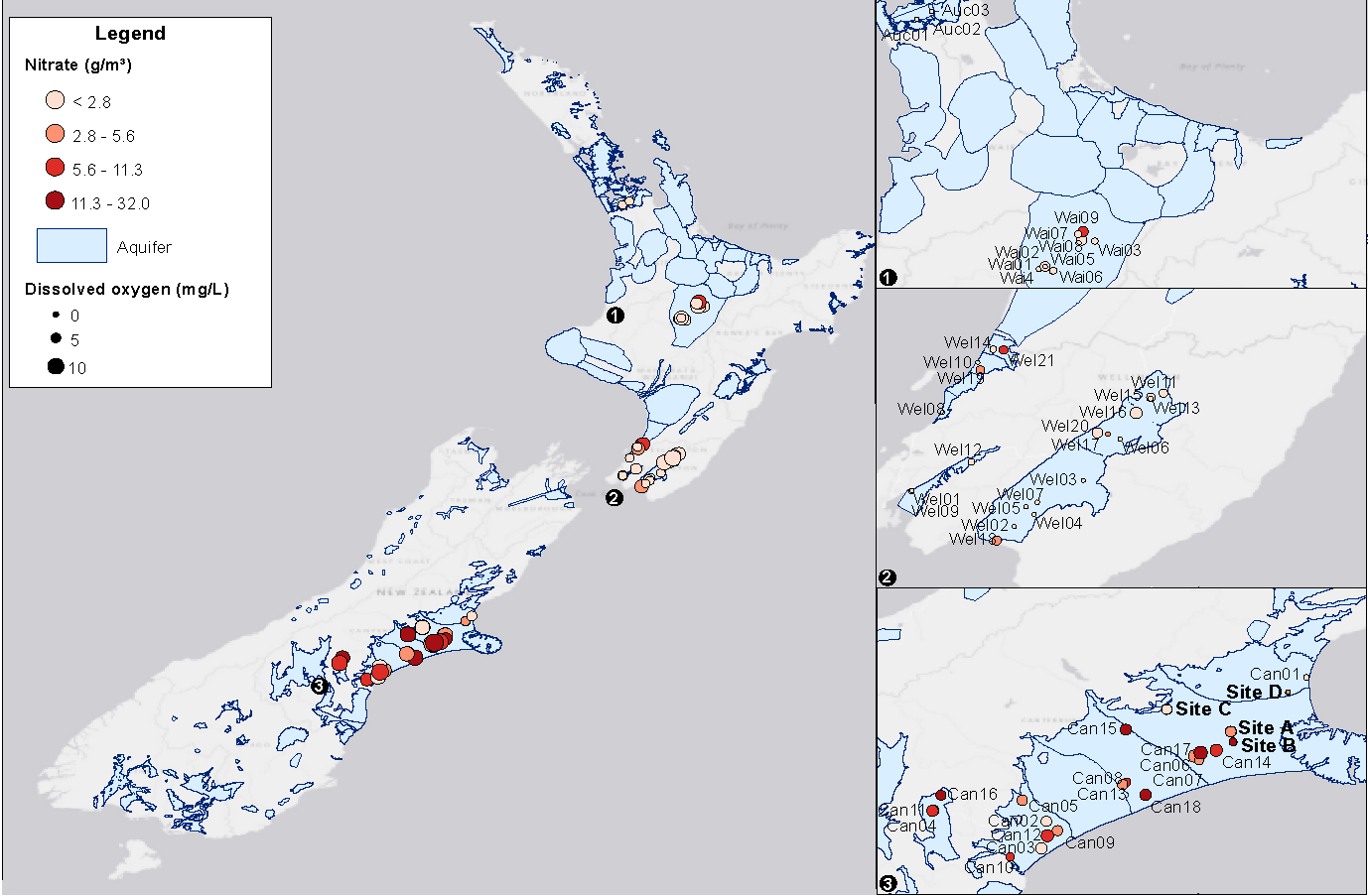


**Figure S1.** Geographic distribution of groundwater sampling sites across New Zealand with corresponding nitrate (red gradient) and oxygen (sized circle) concentrations. 1. Waikato and Auckland 2. Wellington 3. Canterbury. Blue polygons represent aquifers and aquifer boundaries. ArcMap v10.8.1 [17] was used to generate the map using aquifer information and polygon file from https://data.mfe.govt.nz/x/BeFheu (Ministry for the Environment and Statistics, New Zealand). The majority of samples were from alluvial sandy gravel aquifers (50/59 sites), which are a common aquifer type globally [18]. Other aquifer types sampled were volcanic rock (basalt and ignimbrite) and unconsolidated materials such as peat and shellbed. Sampled aquifers constituted a range of nitrate-N (0–22 g/m^3^), DO (0–10.61 mg/L) and DOC (0–26 g/m^3^) concentrations (Table S1), and borehole depths (4.5–28.7m in unconfined, 7.7–114.6m in confined aquifers). Unconfined aquifers are generally more vulnerable to contaminants (e.g. nitrogen) from land use above, and aerobic due to oxygen penetration [36]. In agreement, samples from unconfined aquifers contained higher concentrations of nitrate and DO (Wilcoxon rank, *p* < 0.05).


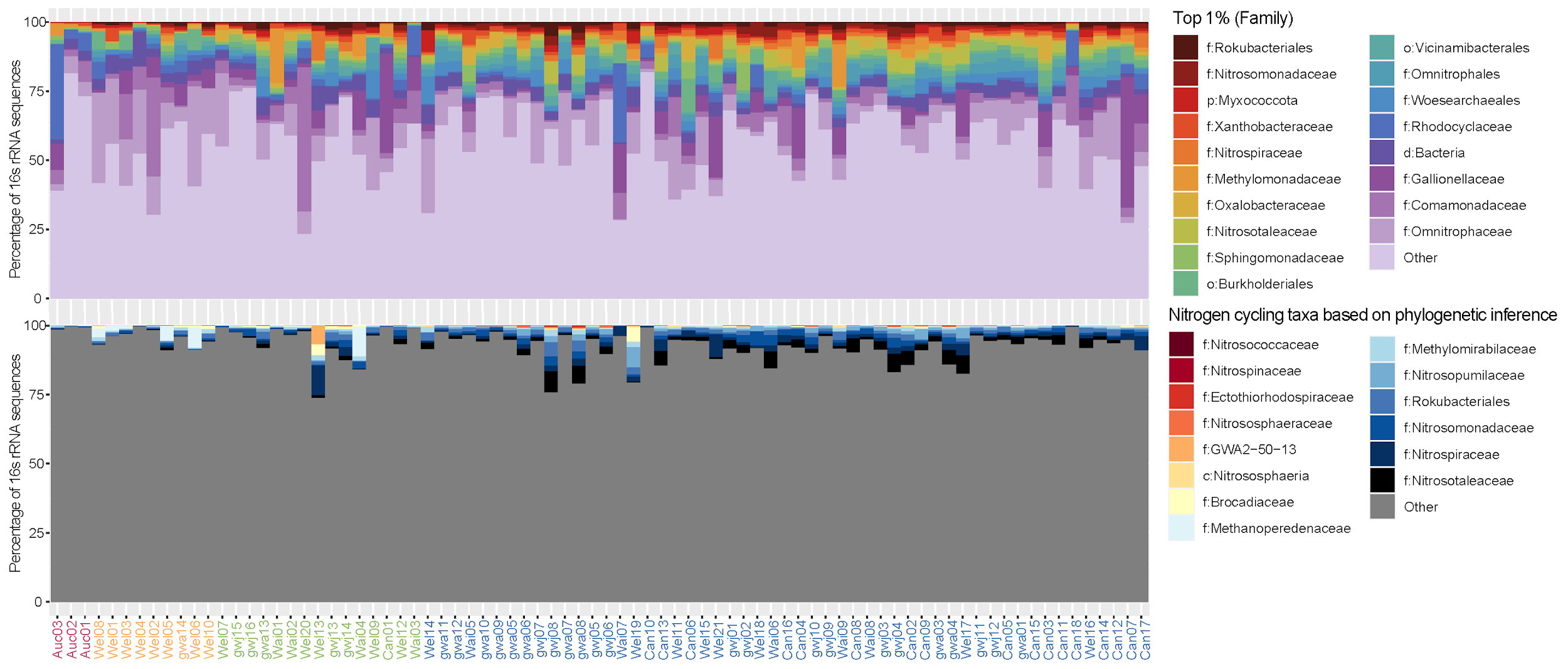


**Figure S2.** Barplots showing the community composition across each well based on amplicon data. (Top) Bar plot showing the community composition for the top 1% of families. (Bottom) Barplot showing families inferred to undertake nitrogen cycling. Sample names are coloured according to oxygen content (Red = Anoxic, Orange = Suboxic, Green = Dysoxic, Blue = Oxic).


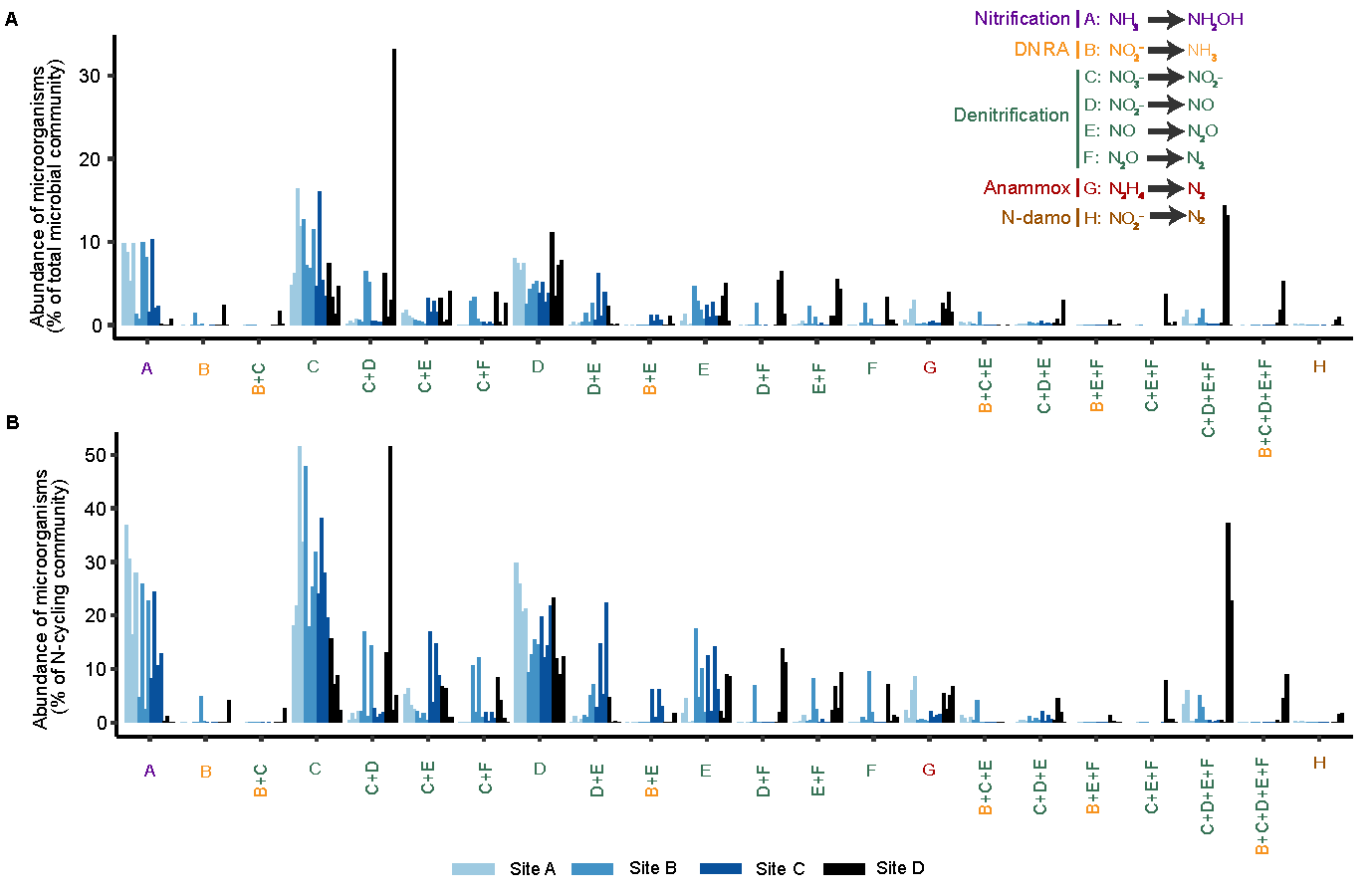


**Figure S3.** The relative abundance of organisms genomically inferred to be involved in one or more nitrogen cycling reactions at sites A-D. **a)** Bar plot showing the relative abundance of MAGs per sample capable of each step in the nitrogen cycle from the total microbial community (MAGs 70-100% complete, 0-5% contamination). **b)** Bar plot showing the relative abundance of nitrogen-cycling MAGs capable of each reaction.


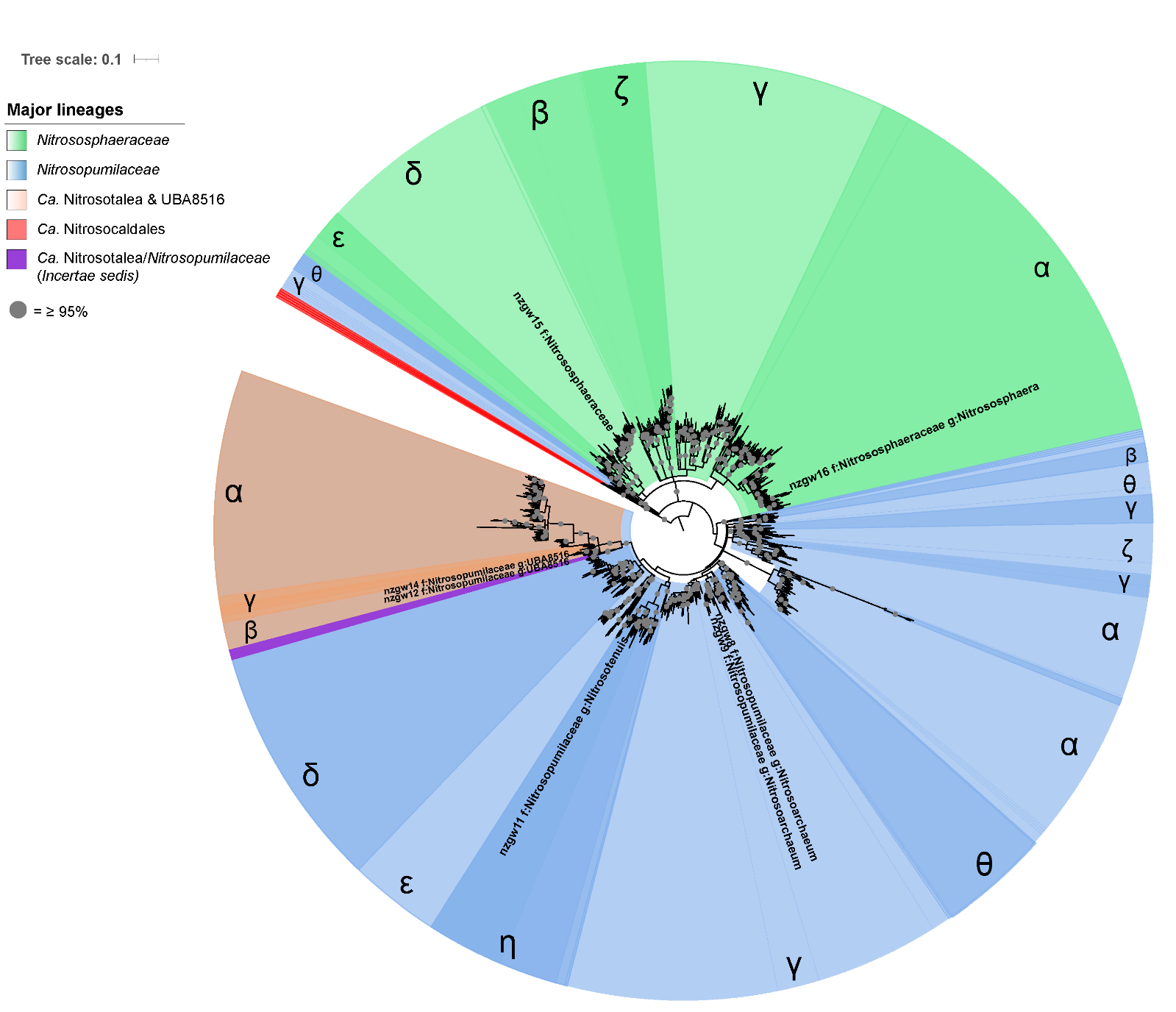


**Figure S4.** Groundwater archaeal *amoA* genes from this study along with global archaeal *amoA* genes from cultivated and environmental AOA. The maximum-likelihood phylogenetic tree was inferred from 1,213 *amoA* genes (598 aligned positions) including 7 *amoA* sequences from this study (shown in bold with the study identifier and GTDB classification given) using *amoA* alignment from Alves et al (2018). The tree shows *amoA* genes clustered with *Nitrosopumilaceae* η (nzgw11) and γ (nzgw8 and nzgw9), *Nitrososphaeraceae* α (nzgw16) and 𝛿 (nzgw15), and *Candidatus* Nitrosotalea β (nzgw12 and nzgw14) sequences. Order-level lineages are indicated by five main different colours, and major constituent subclades are indicated by Greek letters and different shades. The scale bar represents 1 substitution per nucleotide position. The tree was generated by FastTree using GTR model and 1,000 resamples.


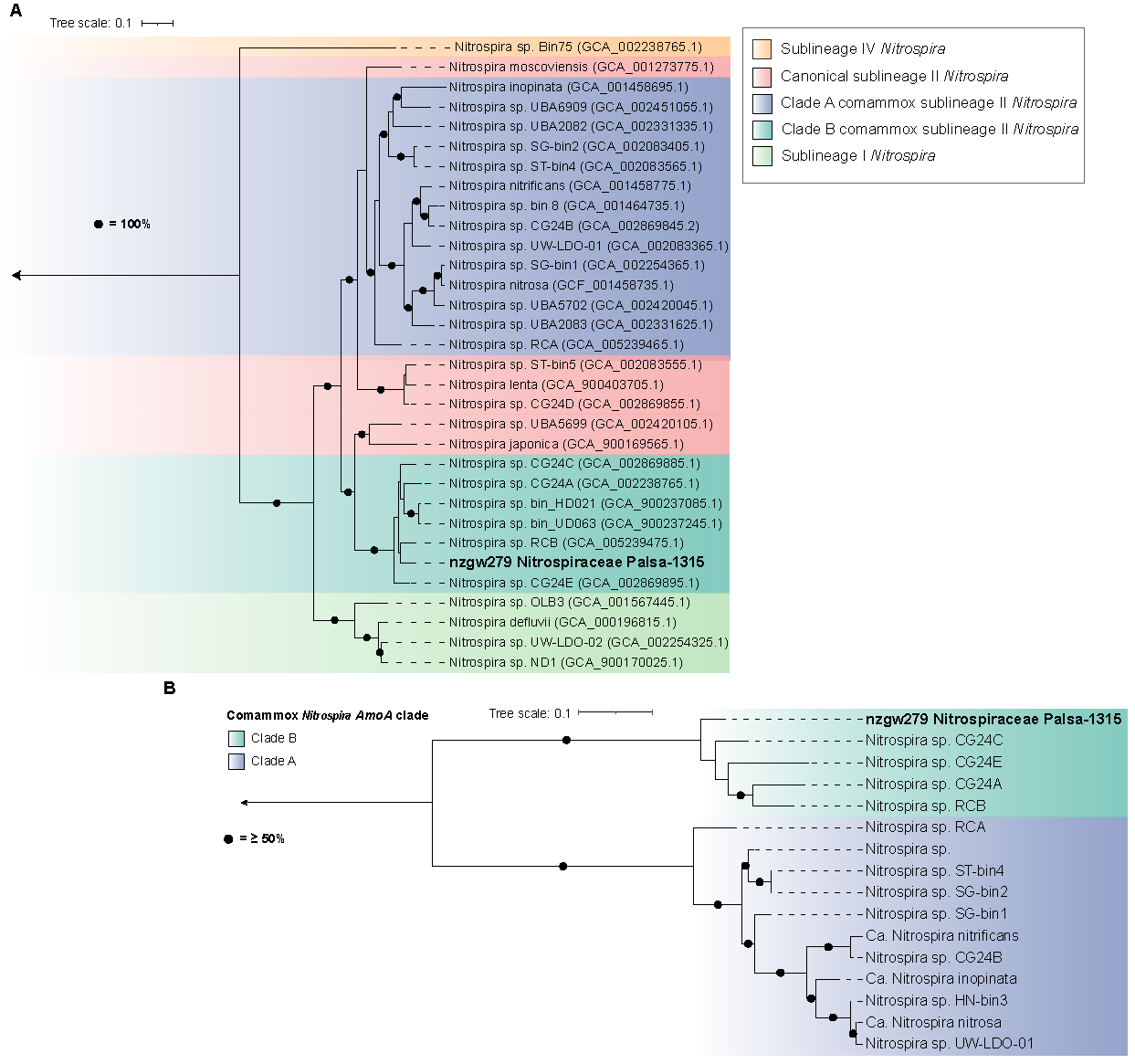


**Figure S5.** Phylogenetic trees representing *Nitrospira* species. **a)** Maximum likelihood phylogenetic tree representing 34 *Nitrospira* genomes based on 120 concatenated bacterial marker genes (GTDB-Tk) with 5,040 amino-acid sites using IQ-TREE best fit model LG+F+G4 and 1,000 bootstraps. Coloured clades represent *Nitrospira* lineages and contain both canonical and comammox. Bootstrap values shown as black circles equal 100%. Scale bar indicates the number of substitutions per site. **b)** Maximum likelihood tree using 18 protein-coding sequences for AmoA with 303 amino-acid sites using LG+G4 model and 1,000 bootstraps. Bootstrap values shown as black circles equal ≥50%. Scale bar indicates the number of substitutions per site. Sequences from this study are shown in bold font, with both the study identifier and GTDB classification given.

**Figure S6.** Maximum likelihood phylogenetic tree constructed from an alignment of *amoA/pmoA* genes (n = 123 sequences) using IQ-TREE best fit model LG+F+I+G4 and 1,000 bootstraps. The sequences from MAGs recovered in this study are coloured red. The *amoA/pmoA* sequences and their annotations were obtained from supplementary data provided by Diamond et al. (2022) [37]. Scale bar represents average changes per amino acid position.
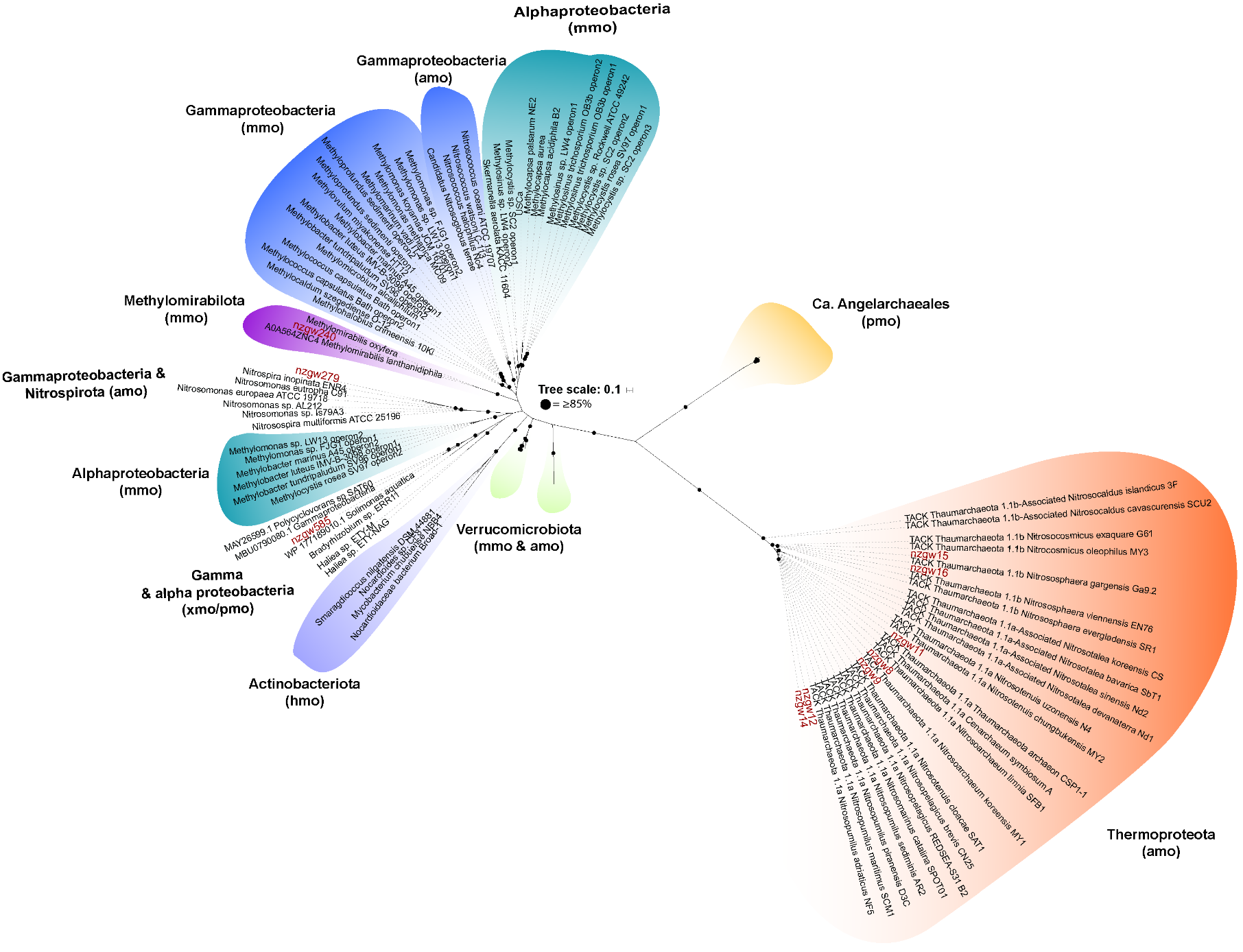


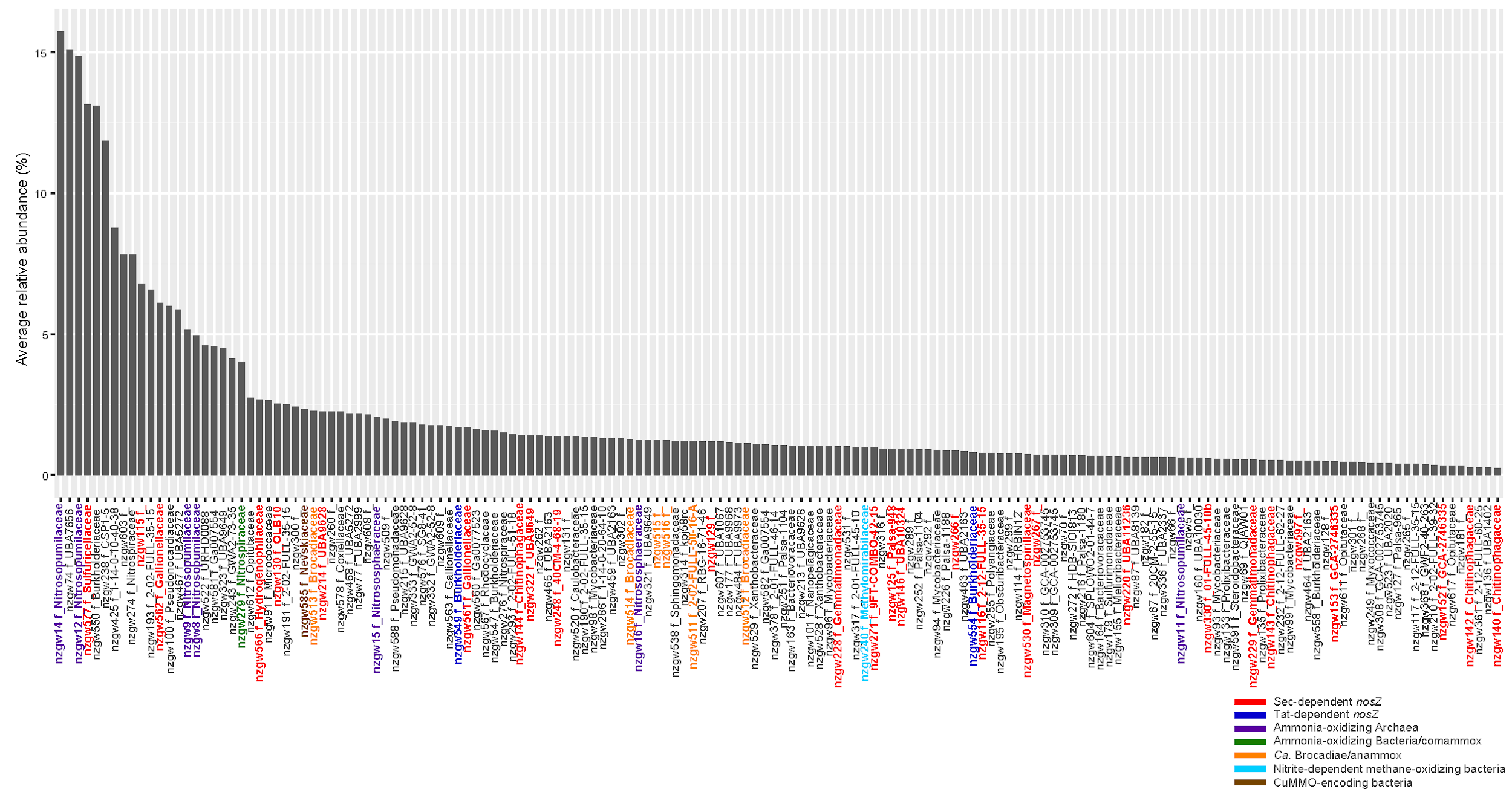


**Figure S7.** Rank abundance curve showing the average relative abundance of each genome that was genomically inferred to perform non-assimilatory nitrogen cycling across sites, labelled with study identifier and family level (f_) classification (GTDB). MAGs have an average relative abundance of 0.3–15.7x (based on normalised coverage) across sites


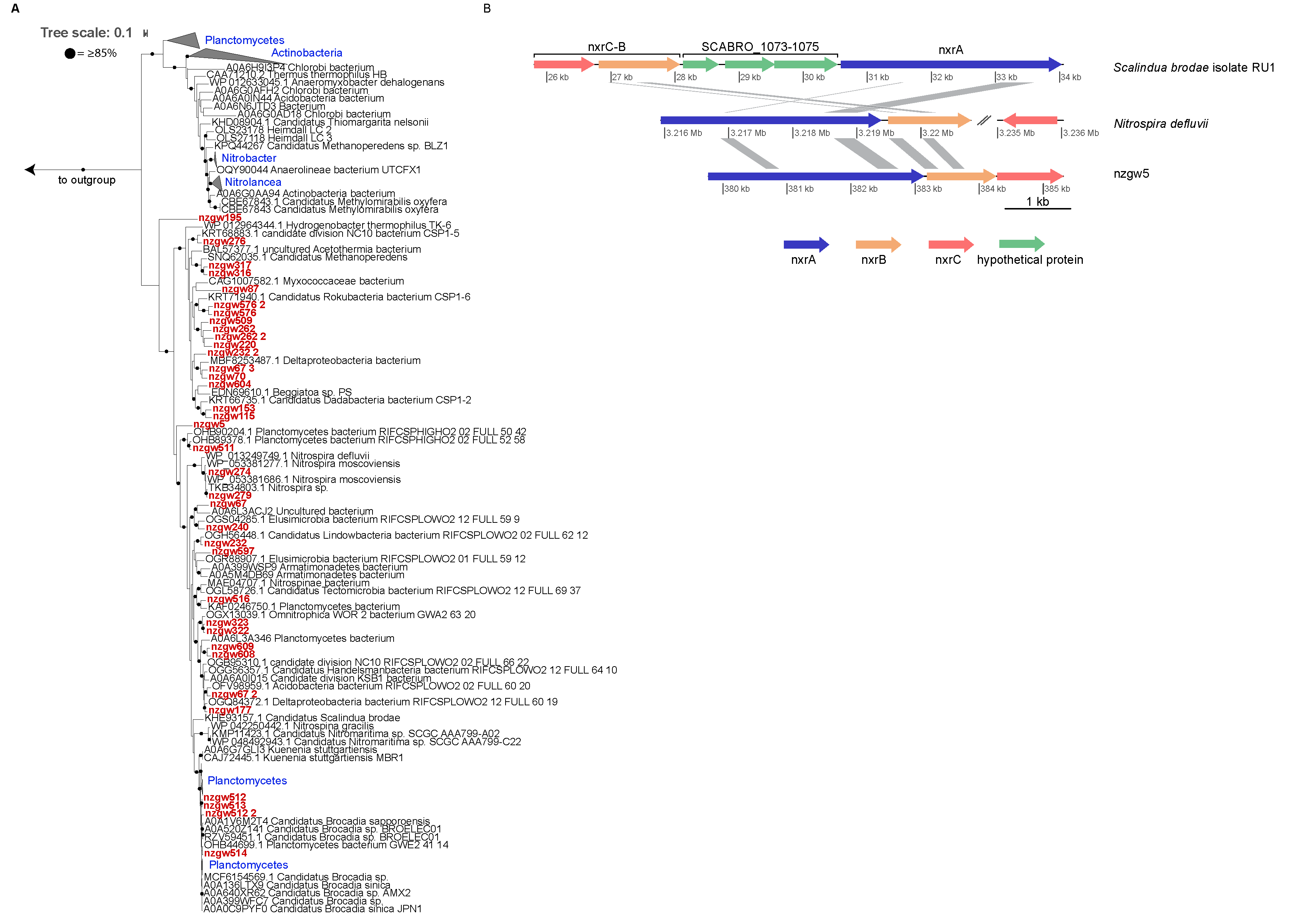


**Figure S8**. Maximum likelihood phylogenetic tree of NxrA protein-coding sequences generated using IQ-TREE and the LG+I+G4 model of substitution and 1,000 bootstraps. Bootstrap values shown as black circles equal ≥85%. The scale bar indicates the number of substitutions per site. Sequences from this study are labelled with study identifier (red font).


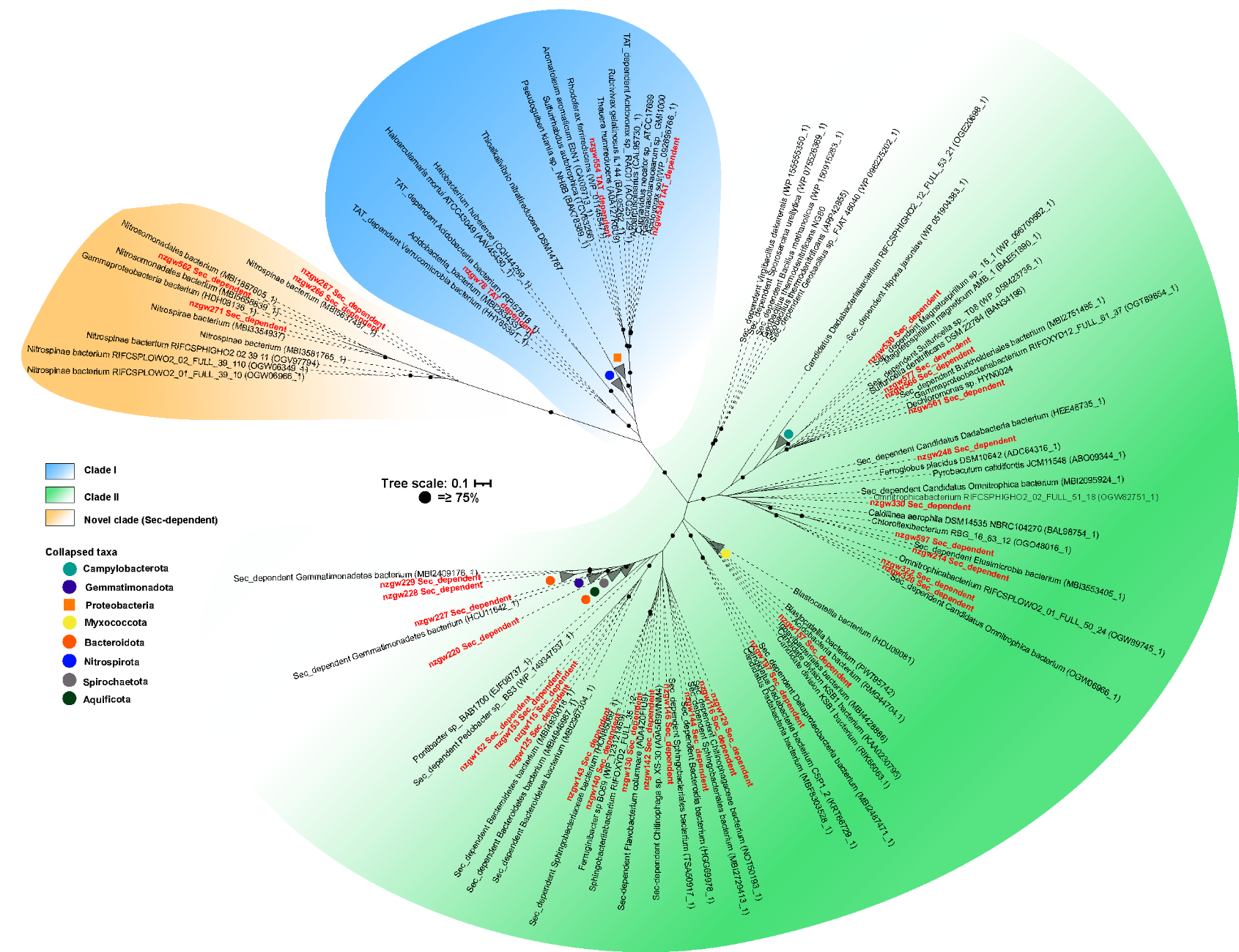


**Figure S9.** Maximum-likelihood phylogenomic tree of 197 NosZ protein-coding sequences generated using IQ-TREE and the LG+I+G4 model of substitution and 1,000 bootstraps. Bootstrap values shown as black circles equal ≥75%. The scale bar indicates the number of substitutions per site. Sequences from this study are labelled with the signal peptide present and study identifier (red font). Sequences were aligned to the secondary structure of *Paracoccus denitrificans* (1FWX) (alignment length = 96 bp) using MUSCLE [38] and trimmed to remove gaps using TrimAL [39].
